# Effect of non-surgical periodontal treatment on cytokines/adipocytokines levels among periodontitis patients with or without obesity: a systematic review and meta-analysis

**DOI:** 10.1186/s12903-023-03383-3

**Published:** 2023-10-05

**Authors:** Yuwei Zhang, Ru Jia, Yifei Zhang, Xuefei Sun, Yukun Mei, Rui Zou, Lin Niu, Shaojie Dong

**Affiliations:** 1 Key Laboratory of Shaanxi Province for Craniofacial Precision Medicine Research, College of Stomatology, Xi’an Jiaotong University, Xi’an, 710004 Shaanxi Province China; 2 Clinical Research Center of Shaanxi Province for Dental and Maxillofacial Diseases, Xi’an, 710004 Shaanxi Province China; 3 Department of Prosthodontics, College of Stomatology, Xi’an Jiaotong University, Xi’an, 710004 Shaanxi Province China

**Keywords:** Systematic review, Obesity, Periodontitis, Non-surgical periodontal therapy, Cytokines, Adipocytokines, Inflammation

## Abstract

**Background:**

The objective of this systematic review and meta-analysis was to evaluate the effects of non-surgical periodontal therapy (NSPT) on inflammatory-related cytokines/adipocytokines in periodontitis patients with or without obesity.

**Methods:**

We followed the preferred reporting items for systematic reviews and meta-analyses statement and registered the study (CRD42022375331) in the Prospective International Register of Systematic Reviews. We screened randomized-controlled trials and controlled clinical trials from six databases up to December 2022. Quality assessment was performed with RoB-2 and ROBINS-I tools for randomized trials and non-randomized trials, respectively. Meta-analysis was carried out using a random-effect model.

**Results:**

We included seventeen references in the systematic analysis, and sixteen in the meta-analysis. Baseline results of pro-inflammatory biomarkers, including serum interleukin (IL)-6, serum and gingival crevicular fluid (GCF), tumor necrosis factor (TNF)-a, serum C-reactive protein (CRP)/hs-CRP, and serum and GCF resistin, were higher in obesity subjects than in normal weight subjects. The effect of NSPT with respect to levels of cytokines/adipocytokines, including IL-6, TNF-a, CRP/hs-CRP, resistin, adiponectin, leptin and retinol binding protein 4 (RBP4), were then analyzed in the systematic and meta-analysis. After three months of NSPT, serum (MD = -0.54, CI = -0.62 – -0.46), and GCF (MD = -2.70, CI = -4.77 – -0.63) levels of IL-6, along with the serum RBP4 (MD = -0.39, CI = -0.68–0.10) decreased in periodontitis individuals with obesity. NSPT also improved GCF adiponectin levels after three months (MD = 2.37, CI = 0.29 – 4.45) in periodontitis individuals without obesity.

**Conclusions:**

Obese status altered the baseline levels of cytokines/adipocytokines (serum IL-6, serum and GCF TNF-a, serum CRP/hs-CRP, and serum and GCF resistin). Then NSPT can shift the levels of specific pro-inflammatory mediators and anti-inflammatory mediators in biological fluids, both in obesity and non-obesity individuals. NSPT can reduce serum and GCF IL-6 levels together with serum RBP4 level in individuals with obesity after 3 months, besides, there is no sufficient evidence to prove that obese patients have a statistically significant decrease in the levels of other cytokines compared to patients with normal weight. NSPT can also increase GCF adiponectin level in normal weight individuals after 3 months. Our findings imply the potential ideal follow-up intervals and sensitive biomarkers for clinical bioanalysis in personalized decision-making of effect of NSPT due to patients’ BMI value.

**Supplementary Information:**

The online version contains supplementary material available at 10.1186/s12903-023-03383-3.

## Background

Periodontitis is a chronic, multifactorial, inflammatory disease related to dysbiotic plaque biofilms and is characterized by progressive destruction of the tooth-supporting apparatus [1–3]. The primary clinical features of periodontitis include the loss of periodontal tissue support, and is typically identified by bleeding on probing, deepened probing depth, loss of attachment, gingival recession, halitosis, and tooth mobility [1, 4]. The present adopted periodontitis classification scheme, unlike the former single category, is based on characterization of the disease due to the multi-dimensional staging and grading system, which is a better assessment for the severity, risk-evaluation, anticipated outcome and management of the disease. Moreover, the current revised classification according to pathophysiology is categorized as periodontitis, necrotizing periodontitis and the one as the direct manifestation of systemic diseases [1]. Periodontitis is associated with the elevation of inflammatory molecules in systemic diseases and the development of several systemic co-morbidities, such as cardiovascular disease and type 2 diabetes [5, 6]. Obesity, which manifested as the accumulation of excess body fat, is another common health concern that results in a significant economic and societal burden worldwide. WHO currently accepts a body mass index of 25 kg/m^2^ or greater as abnormal and when BMI is 30 kg/m^2^ or over the objects are categorized as obese [7, 8]. Furthermore, obesity is currently recognized as a state of low-grade systemic inflammation (LGSI) [9]. Research has demonstrated negative associations between bone metabolism, periodontitis, and obesity. Clinical studies have found that obesity is correlated with reduced bone mass, which can lead to varying degrees of osteoporosis and alveolar bone resorption as present in periodontitis [7, 10]. Obese individuals also tend to have higher levels of serum inflammatory biomarkers, such as cytokines (adipokines), which are secreted from adipocytes in adipose tissues. These biomarkers include interleukins (IL-6 and IL-8), tumor necrosis factor α (TNF-α), interferon-γ (IFN-γ), C-reactive protein (CRP), monocyte chemoattractant protein-1 (MCP-1), chemerin, adiponectin, omentin, isthmin 1, nesfatin-1, leptin, retinol-binding protein 4 (RBP4), resistin, and visfatin. They modulate inflammatory, immune, and metabolic responses [11–14].

Studies have also demonstrated that the levels of the inflammatory cytokines or adipokines mentioned above exhibit varying trends of change in patients with periodontitis at different stages of the disease. The association between periodontal disease and obesity was initially discovered in obese Zucker rats by Perlstein and Bissada [15]. Moreover, recent studies and reviews have further revealed the association between obesity and periodontal disease [16–18]. Additionally, periodontal disease and obesity/overweight status may have a bidirectional relationship, to be specific, not only are obese/overweight individuals prone to periodontal disease, but also periodontal disease may exacerbate dyslipidemia [19–21]. In fact, during the local and systemic comorbid pathological condition, both periodontal tissues and adipocytes as host immune responsive cells, when triggered by pathogens, can secret higher levels of pro-inflammatory cytokines in obese individuals (such as IL-1, IL-1β, IL-6, TNF-a, etc.) to exacerbate both disease conditions [22]. Also, obesity is related to the increased susceptibility towards bacterial infection; while periodontal tissues affected by periodontal disease (a source of bacteremia) may potentially worsen the obesity condition via pathways that involve the enhanced generation of reactive oxygen species (ROS) [23, 24]. And the recent discovery revealed that the association between periodontal disease and obesity is the outcome of an overall imbalance between health and systemic health [16].

Non-surgical periodontal therapy (NSPT) remains the gold standard for managing chronic periodontitis. The removal of supragingival plaque and subgingival scaling and root planning is the gold standard non-surgical therapy. Nevertheless, the mere mechanical debridement may not eradicate all subgingival periopathogens, and the adjunctive non-surgical measures are sometimes utilized to eliminate bacterial biofilm and deposits and restore a balanced microbiota environment for periodontal health [25]. NSPT also reduces local inflammatory stimulation, decreases pocket depth, and promotes the recovery of clinical attachment by restoring highly perfused and collagen-rich connective tissues. As there is a correlation between periodontal disease and obesity, exploring the effectiveness of NSPT for obese patients with periodontitis is reasonable [26–29].

The 2017 World Workshop had demonstrated one of the issues that should be addressed in the future research was the identification of microbial, genetic, or host response‐associated biomarkers that discriminate between the periodontitis phenotypes, also the ones that can reflect whether the initiation or progression of periodontitis [1]. As far as we concern, the aforementioned cytokines seem to get involved in the mechanisms of periodontitis and obesity comorbid condition, and the levels of those cytokines before and after the NSPT are expected to provide the adjunctive measurements for the effect of the therapy and the primary prediction the role of these markers involved in the recovery of the comorbid. However, limited studies or weak evidence make it unclear whether NSPT significantly impacts clinical periodontal outcomes in obese individuals compared to non-obese individuals. Additionally, the role of related cytokines or adipokines before and after NSPT, as well as their impact on treatment outcomes, remains unclear. Therefore, the purpose of this study is to systematically review the efficacy of NSPT in managing periodontitis in both obese and non-obese individuals while considering the levels of biomarkers.

## Materials and methods

### Protocol and registration

This systematic review and meta-analysis were carried out in accordance with the preferred reporting items for systematic reviews and meta-analyses (PRISMA) guidelines [30] and registered in the Prospective International Register of Systematic Reviews (PROSPERO), with approval from all authors (CRD42022375331).

### Focused question and selection criteria

One specific clinical focused question was constructed according to the Participants, Interventions, Control, and Outcomes (PICO) principle [31]: “what is the efficacy of NSPT with respect to cytokine/adipocytokine levels in obese individuals? Does NSPT reduce their biofluid levels of related cytokines/ adipocytokines (IL-6, TNF-a, CRP/hs-CRP, resistin, adiponectin, leptin, and RBP4)?

- (P) Participants: individuals with obesity and periodontitis
- (I) Types of intervention: NSPT
- (C) Comparison: without periodontal treatment
- (O) Outcome: biofluid levels of related cytokines/ adipocytokines

The secondary focused question was: “what is the efficacy of NSPT with respect to cytokine/ adipocytokine levels in obese individuals compared to non-obese chronic periodontitis patients? Are obese people more responsive to treatment in cytokines/ adipocytokines levels?”

- (P) Participants: individuals with obesity and periodontitis
- (I) Types of intervention: NSPT
- (C) Control intervention: NSPT in non-obese patients with chronic periodontitis
- (O) Outcome measures: biofluid levels of cytokines/adipocytokines from measured from the baseline to the follow-up

Studies were considered eligible once they reached the following criteria: (1) Randomized clinical trials (RCTs) were deemed the most appropriate study design, along with controlled clinical trials (CCTs), for evaluating the efficacy of NSPT on cytokine/adipocytokine levels in individuals with or without obesity. (2) the study reported the association of given cytokines/ adipocytokines level with patients affected by obesity and the control group was necessary; (3) NSPT should be applied; (4) full-text were published in English. Studies were excluded once they were (1) laboratory animal studies, case–control studies, cross-sectional studies, case series, systematic reviews, literature reviews, conference abstracts; (2) studies lacking complete data on levels of targeted cytokines/adipocytokines (despite efforts to retrieve raw values from the original authors); (3) clinical analyses involving periodontitis patients with systemic conditions other than obesity or who received treatments beyond NSPT were excluded. Please refer to Table S1 for details on the inclusion and exclusion criteria.

### Criteria for obesity and periodontal diagnosis

The WHO definition of obesity in adults considers measurements such as waist circumference, waist-hip ratio, and body fat [32]; we followed the WHO obesity BMI diagnostic criterion (Table 1). According to WHO criteria, individuals with BMI over 30 kg/m^2^ are categorized as obese [7], and BMI over 27.5 kg/m^2^ is the recommended BMI cut-off point for obesity in Asian populations according to the interpretation of WHO expert consultation [33]. Previous studies measured metabolic [29, 34–41] and anthropometric [34–37, 41–45] parameters following NSPT to determine whether participants remained classified as obese during follow-up, particularly for longer-term studies. Various definitions and criteria for periodontitis were used by different authors and were accepted for the purposes of this review. The NSPT interventions included professional oral hygiene instructions, full-mouth scaling and root planning (SRP), or SRP combined with local or systemic antimicrobial agents. No surgical procedures, such as periodontal flap surgery, were involved in the NSPT.

**Table 1:** Characteristics of the included studies in the systematic review

Study	Design; setting	Participants (Sample size; mean age;characteristics; sex; dropouts	Periodontal criterionfor inclusion	Obesity diagnostic criterion	Periodontal Interven-tion			Follow-up				
								Time	Periodontal	Biomarker	Metabolic	Anthropometric
Al-Hamoudi et al., 2017 [46]	Parallel arm, prospective CCT; Riyadh, Saudi Arabia	**G1: OP** 35/39.5yrs/BMI 39.2 **G2: NP** 35/36.3yrs/BMI 21.6 ^a^**G3: O** 34/37.5yrs/BMI 33.6 ^a^**G4: N** 33/36.2yrs/BMI 22.4 M/F 135/2 dropouts: 0	BOP, PD ≥ 4 mm, MBL ≥ 3 mm in more than 30% of sites	BMI ≥ 27.5 kg/m^2^	SRP; OHI			6 mon	BOP; PD; MT; MBL	Whole salivary **resistin** G1:T0 = 18.4; T1 = 9.8; G2:T0 = 20.5; T1 = 8.6 ng/mL; **IL-6** G1:T0 = 14.3; T1 = 7.5; G2:T0 = 15. 1; T1 = 5.8 pg/mL	NR	NR
Zuza et al.,2011 [20]	CCT; Barretos (UNIFEB), Barretos, Brazil	**G1: OP** 27/45. 1yrs/BMI 35.3 **G2: NP** 25/42.9yrs/BMI 23.0 M/F 14/38 dropouts: 0	Clinical diagnosis of generalized chronic periodontitis	BMI ≥ 30 kg/m^2^, WHR ≥ 0.8(F) and ≥ 0.9(M); WC > 88 cm(F) and > 102 cm(M); BF ≥ 33%(F) and ≥ 25%(M)	SRP; OHI; plaque control			3 mon	VPI, GBI, BOP, PPD, and CAL	Serum: **IL-1β** G1:T0 = 2.44; T1 = 1.35; G2:T0=1.62; T1=0.90; **IL-6** G1:T0 = 1.84; T1 = 1.29; G2:T0 = 1. 14; T1 = 0.57; **TNF-a** G1:T0 = 22.29; T1 = 8.52; G2:T0 = 17.43; T1 = 5.23; **IFN-γ** G1:T0 = 0. 15; T1 = 0.015; G2:T0 = 0. 14; T1 = 0.019 pg/mL	Fasting glucose, glycated hemoglobin	BMI, WC, WHR, and BF
Vohra et al.,2018 [47]	Parallel-arm, randomized CCT; Riyadh Saudi Arabia	**G1: OP + PDT** 23/51.84yrs/BMI 34.8 **G2: OP** 29/48.68yrs/BMI 31.4 M/F 44/8 dropouts: 0	BOP, PD ≥ 4 mm, MBL ≥ 3 mm in more than 30% of sites	BMI ≥ 27.5 kg/m^2^	SRP with or without aPDT; OHI			12 weeks	PI; BOP; PD and CAL	GCF: **TNF-a** G1:T0 = 13. 12; T1 = 2. 17; G2:T0=13.34; T1=2.97; **IL-6** G1:T0 = 8.74; T1 = 3.09; G2:T0 = 8.58; T1 = 4. 17 pg/mL;	NR	NR
TED et al., 2015 [37]	CCT; Guarulhos, Sao Paulo, Brazil	**G1: OP** 20/50.0yrs/BMI 36.1 **G2: NP** 20/48.5yrs/BMI 23.4 M/F 21/19 dropouts: 0	> 30% of the sites with concomitant PD and CAL ≥ 4 mm and a minimum of six teeth distributed in the different quadrants presenting at least one site with PD and CAL ≥ 5 mm	BMI ≥ 30 and < 40 kg/m^2^ and concomitant WHR ≥ 0.85(F)and WHR ≥ 0.90(M)	OHI; supragingival plaque and calculus removal, exodontia, provisional restoration; SRP			3 mon 6 mon 12 mon	PI; BOP; PD and CAL	Serum and GCF: **resistin**; **adiponectin**; **leptin**; **TNF-a** and **IL- 6**. There were no changes in serum levels of any adipokines for any group after therapy (p > 0.05). Patients with obesity exhibited higher serum levels of leptin at all time-points and IL-6 at 3 months post-therapy (*p* < 0.05)	NR	BMI, WHR
Akram et al.,2017 [34]	Parallel-arm, randomized CCT; Kuala Lumpur, Malaysia	**G1: OP + NSPT** 33/44.68yrs/BMI 32.98 ^a^**G2: OP** 33/44.84yrs/BMI 35.83 M/F 17/45 dropouts: 4	≥ 2 interproximal sites with CAL ≥ 4 mm [not for the same tooth] or ≥ 2 interproximal sites with PPD ≥ 5 mm [not for the same tooth] ≥ 3 mm of clinical attachment loss at ≥ 30 of the sites	BMI ≥ 27.5 kg/m^2^ and concomitant WHR ≥0.85(F)and WHR ≥ 0.90(M)	SRP; OHI and 0. 12% 6 weeks chlorhexidine 12 weeks gluconate mouthwash				PS; GBI; CAL; PD	Salivary: **resistin** G1:T0 = 12.26; T1(12 weeks) = 11.62 ng/mL	NR	NR
Al-Zahrani and Alghamdi, 2012 [48]	CCT; Jeddah; Saudi Arabia	**G1: OP** 20/44.0yrs/BMI NR **G2: NP** 20/43.4yrs/BMI NR M/F 0/40 dropouts: 0		BMI ≥ 30 kg/m^2^	SRP; 2 mon OHI				CAL; PD; BOP; PS	Serum **CRP** G1:T0 = 0.96; T1 = 0.79; G2:T0 = 0.60; T1 = 0.28 mg/l	NR	NR
Md Tahir et al., 2020 [49]	Prospective CCT; Kuala Lumpur, Malaysia	**G1: OP** 18/44.7yrs/BMI 33.3 **G2: NP** 30/47.2yrs/BMI 23.8 M/F 23/25 outlier:6 dropouts: 0	Eke et al. [50]	BMI ≥ 30 kg/m^2^	SRP; OHI and 0. 12% chlorhexidine gluconate mouthwash	12 weeks			CAL; PD; VPI and GBI	Serum: **resistin** G1:T0 = 14.7; T1 = 17.6; G2:T0 = 6.9; T1 = 9.5 ng/mL	NR	NR
Duzagac E et al., 2016 [30]	CCT; Istanbul, Turkey	**G1: OP** 15/40.66yrs/BMI 36.26 **G2: NP** 15/41.06yrs/BMI 22.14 ^a^**G3: N** 15/39.66yrs/BMI 21.71 M/F 19/26 dropouts: 0	PB ≥ 4 mm in ≥ 30% of periodontal Sites;BOP in ≥ 50% of sites; interproximal CAL > 2 mm in ≥ 20% of periodontal sites; and radiographic evidence of bone loss	BMI ≥ 30% and simultaneous WHR > 0.95(M) and 0.80(F)	SRP; 3 mon OHI				PI; GI; BOP; CAL; PD	Serum and GCF: **adiponectin; IL-6; TNF-a; IL-10** and serum **CRP** level. OP and NP patients responded to NSPT with consistent adipokine levels in GCF. However, obesity affects the CRP and serum adipocytokine levels in response to therapy	Serum HDL, LDL, TGR	BMI; WHR
ÇETİNER et al., 2018 [31]	CCT; Ankara, Turkey	**G1: OP** 31/45.26yrs/BMI 34.76 **G2: NP** 19/35.58yrs/BMI 23.53 M/F 10/40 dropouts: 0	≥ 30% of the sites with bone loss, and at least two non-adjacent teeth with ≥ 1 sites with PD ≥ 5 mm and CAL ≥ 5 mm in each quadrant, BOP and radiographic evidence of bone loss	BMI ≥ 30 kg/m^2^ and WC > 88 cm (F) and > 102 cm (M)	SRP; 3 mon OHI				PI; GI; PD; CAL; BOP	GCF: **TNF-a** G1:T0 = 8.42; T1 = 6.56; G2:T0=9.66; T1=9.06 **IL-6** G1:T0 = 2.87; T1 = 1.45; G2:T0 = 0.81; T1 = 0.73 **Visfatin** G1:T0 = 11.71; T1 = 6.83; G2:T0 = 8.81; T1 = 7.44 pg	TC; TGR; HDL; LDL	BMI; WC
Suresh et al., 2018 [26]	CCT; Tamil Nadu, India	**G1: OP** 30/45.26yrs/BMI 34.76 **G2: NP** 30/35.58yrs/BMI 23.53 M/F 10/40 dropouts: 0	CAL ≥ 3 mm in more than 30% of sites	BMI ≥ 30 kg/m^2^ and WC > 88 cm (F) and > 102 cm (M)	supragingival 2 mon Scaling; SRP				GI; CAL; PPD	GCF **resistin** G1:T0 = 15.06; T1 = 12.33; G2:T0 = 12.83; T1 = 6.97 and serum **resistin** G1:T0 = 25.83; T1 = 25.81; G2:T0 = 19.07; T1 = 15.72 ng/mL	plasma ROM levels	NR
Zuza et al., 2016 [32]	CCT; Barretos, Brazil	**G1: OP** 28/45.7yrs/BMI 35.3 **G2: NP** 26/42.7yrs/BMI 23.0 M/F 14/40 dropouts: 0	At least six teeth with PD ≥5 mm and CAL ≥ 3 mm, and BOP All more than 30% of sites	BMI ≥ 30 kg/m^2^ and WHR ≥ 0.85(F) and WHR ≥ 0.90(M); WC > 88 cm (F) and > 102 cm (M); the percentage of BF ≥ 35%(F) and ≥ 25% (M)	Supra and subgingival 3 mon scaling and root planning; OHI				PI; GBI; PD; CAL and BOP	Serum hs-CRP G1:T0 = 3.75; T1 = 2.62; G2:T0 = 1.45; T1 = 1.01 mg/ dL	TGR, TC, HDL, LDL, fasting glucose, glycated hemoglobin	BMO; WC; BF
Martinez-Herrera et al., 2018 [33]	CCT; Valencia, Spain	**G1: OP** 96/42.7yrs/BMI 42.5 **G2: NP** 48/39.4yrs/BMI 22.7 ^a^**G3: O** 23/42.2yrs/BMI 39.6 ^a^**G4: N** 64/36.8yrs/BMI 21.8 M/F 64/167 dropouts: 37	Four or more teeth had one or more sites with PD ≥ 4 mm and CAL ≥ 3 mm	BMI ≥ 30 kg/m^2^	OHI; Supra and subgingival scaling and root planning; chlorhexidine mouthwash (0. 12%)	3 mon			PD; CAL; BOP	serum: **TNF-a** G1:T0 = 17.23; T1 = 13.90; G2:T0 = 10.8; T1 = 9.65 pg/ml **IL-6** G1:T0 = 3.79; T1 = 3.38; G2:T0=4.55; T1=4.59 pg/ml **hs-CRP** G1:T0 = 8.33; T1 = 8.57; G2:T0 = 2.46; T1 = 1.68 mg/L and **RBP4** G1:T0 = 3.84; T1 = 3.46; G2:T0 = 3.20; T1 = 3.02 mg/dl	glucose, TC; TGR; HDL; LDL	BMI; WHR
Herrera et al., 2020 [39]	A Pilot RCT Study; Valencia, Spain	**G1: OP** 23/43.6yrs/BMI 42.3 ^a^**G2: OP** 26/43.8yrs/BMI 44.6 M/F 64/167 dropouts: 3	Interdental CAL ≥ 2 non-adjacent teeth, or buccal or oral CAL ≥ 3 mm with pocketing > 3 mm detectable at ≥ 2 teeth	BMI ≥ 30 kg/m^2^	OHI; Supra and subgingival scaling and root planning; chlorhexidine mouthwash (0. 12%)	12 weeks			PD; CAL; BOP	Serum **TNF-a** G1:T0 = 17.8; T1 = 16.5 pg/dl; **hs-CRP** G1: T0 = 4.67; T1 = 5.85 mg/L; **RBP4** G1: T0 = 4.38; T1 = 3.95 mg/dl	Fasting glucose; Insulin TC; TGR; HDL; LDL	BMI; waist
Goncxalves et al., 2015 [40]	CCT; Sa˜o Paulo, Brazil	**G1: OP** 24/48.8yrs/BMI 33.2 **G2: NP** 24/48.4yrs/BMI 24.4 M/F 26/13 dropouts: 9	> 30% of the sites with concomitant PD and CAL ≥ 4 mm and a minimum of six teeth distributed in the different quadrants presenting at least one site	BMI ≥ 30 and <40 kg/m2 and concomitant WHR	OHI; supragingival plaque and calculus removal,	3 mon 6 mon			PD; CAL; BOP; MB; suppuration	Serum **leptin** G1:T0 = 441.8; T1(3 m) = 475.7 T2(6 m) = 421.8; G2:T0 = 292.7; T1(3 m) = 307.7 T2(6 m) = 297.4 (10^2^ pg/ml)	NR	BMI; WHR
			with PD and CAL ≥ 5 mm and BOP	≥ 0.85(F)and WHR ≥ 0.90(M)	exodontia, provisional restoration; SRP					and **adiponectin** G1:T0 = 52.5; T1(3 m) = 49.1 T2(6 m) = 47.4; G2:T0 = 44.2; T1(3 m) = 41.2 T2(6 m) = 48.2 (10^2^ ng/ml)		
Altay et al., 2013 [35]	CCT; Ankara, Turkey	**G1: OP** 22/45.6yrs/BMI 32.2 **G2: NP** 24/42.5yrs/BMI 26.3 M/F 14/32 dropouts: 0	The presence of ≥ 5 teeth with ≥ 1 sites with PD ≥ 5 mm, CAL ≥ 2 mm, and positive on BOP	BMI ≥ 30 kg/m^2^ and WC > 88 cm (F) and > 102 cm (M)	SRP; OHI; chlorhexidine 0. 12% Solution and gel		3 mon		PI; GI; PD; BOP; CAL	Serum **TNF-a** G1:T0 = 5.8665; T1 = 3.9101; G2:T0 = 3.8791; T1 = 3.2791 mg/L **IL-6** G1:T0 = 1.2794; T1 = 0.7794; G2:T0 = 1. 1074; T1 = 0.7865 ng/L **leptin** G1:T0 = 22.2011; T1 = 17.9169; G2:T0 = 16.4468; T1 = 13.073 ng/L **hs-** **CRP** G1:T0 = 4.233; T1 = 3.423; G2:T0 = 3.5149; T1 = 3.3149 mg/L	TGR; TCHDL; LDL; insulin; lipoprotein; a fasting blood glucose	NR
Suvan et al., 2021 [36]	CCT with cohort observation; London, UK	**G1: OP** 58/47yrs/BMI 35.58 **G2: NP** 57/50yrs/BMI 23.10 M/F 52/63 dropouts: 0	Probing pocket depths of ≥ 5 mm and marginal alveolar bone loss with > 30% sites affected	BMI ≥ 30 kg/m^2^	Full mouth mechanical periodontal debridement; OHI		2 mon 6 mon		PD; CAL; full- mouth bleeding score and plaque score	Serum **hs-CRP** G1:T0 = 4.0887; T1(2 m) = 3.574 T2(6 m) = 3.2974; G2:T0 = 0.7414; T1(3 m) = 0.706 T2(6 m) = 0.8767 mg/L	TGR; TCHDL; LDL; fasting blood glucose; insulin; malondialde hyde	BMI
Abdellatif et al., 2022 [51]	RCT; Karnataka, India	**G1: OP** 40/48.5yrs/BMI 36.2 ^a^**G2: O** 40/47. 1yrs/BMI 35.8 **G3: NP** 42/51.2yrs/BMI 19.07 Papapanou et al. [1] BMI ≥ 30 kg/m^2^ ^a^**G4: N** 41/47.2yrs/BMI 20.2 M/F 119/44 dropouts: 0			Scaling and root planing and 3 mon photodynamic therapy				PI; BI; PB; CAL; MBL; number of missing teeth	Salivary **leptin** G1:T0(SRP + PDT) = 265.7; T1(SRP + PDT) = 210.2; T0(SRP) = 246.4; T1(SRP) = 217.3 G3:T0(SRP + PDT) = 121.4; T1(SRP + PDT) = 51.2; T0(SRP) = 117.7; T1(SRP) = 41.2 pg/ml **Adiponectin** G1:T0(SRP + PDT) = 3.43; T1(SRP + PDT) = 3.21; T0(SRP) = 3.82; T1(SRP) = 3.14 G3:T0(SRP + PDT) = 3. 15; T1(SRP + PDT) = 2.5; T0(SRP) = 3.3; T1(SRP) = 2.88 μg/ml	NR	NR

^a^ Groups that not take into account; *T0* initial examination, *T1* examination after periodontal therapy, *OP* obesity with periodontitis, *NP* normal weight with periodontitis, *O* obesity, *N* normal weight, *mon* month, *BMI* body mass index (kg/m^2^), *MBL* marginal bone loss, *MT* missing teeth, *BF* body fat, *WHR* waist-hip ratio, *WC* waist circumference (cm), *OHI *oral hygiene instructions, *aPDT* antimicrobial photodynamic therapy, *PS* plaque score, *GBI* gingival bleeding index, *NR* not reported, *VPI* full mouth visible plaque index, *GBI* gingival bleeding index, *PI* plaque index, *GI* gingival index, *HDL* high-density lipoprotein, *LDL* low-density lipoprotein, triglyceride, *ROM* plasma-reactive oxygen metabolite, *TGR* triglycerides (mg/dL), *TC* total cholesterol (mg/dL), *CRP* C-reactive protein (mg/dL), *hs-CRP* high-sensitivity C-reactive protein (mg/dL), *TG* triglycerides, *MB* marginal bleeding, *F* female, *M* male

### Information sources and search protocol

Two reviewers, Z. YW and Z. YF, conducted a systematic search for articles addressing the research question in English from 1977 to November 2022. They searched six electronic databases, including PubMed, ISI Web of Knowledge, ScienceDirect, Web of Science, Scopus, and OpenGrey, and limited the searches to the first 200 hits in Google Scholar. In addition, a manual search of the relevant literature was performed. The search strategy included keywords related to ((periodontal disease OR periodontitis OR chronic periodontitis OR adult periodontitis OR attachment loss OR aggressive periodontitis OR juvenile periodontitis) AND (root planning OR periodontal treatment OR non-surgical periodontal therapy) AND (obesity OR obese OR body mass index) AND (cytokine OR adipocytokine OR IL-6 OR TNF-a OR CRP OR hs-CRP OR resistin OR adiponectin OR leptin OR RBP4)). Any disagreements between the two reviewers were resolved by a third author, J. R.

### Study selection

The reviews screened titles and abstracts based on the inclusion and exclusion criteria. Full-text articles were obtained for references that did not provide sufficient information for inclusion or exclusion based on their titles and abstracts. After removing duplicates, all articles that met the eligibility criteria were included. Any disagreements between the two reviewers were resolved through discussion and consensus with a third author (J. R). The Kappa score for this stage was 0.79, indicating substantial agreement.

### Data extraction and quality assessment

The same two reviewers undertook data extraction independently. Information from the accepted studies was tabulated according to the author(s), publication year, study designs, settings, participant characteristics, sample characteristics, periodontal and obesity criteria for inclusion, and follow-up outcomes with cytokines/adipocytokines investigated. Data collection was based on the focused question addressed in the systematic review. Data on baseline and post-treatment follow-up timepoints that compared the levels of cytokines/ adipocytokines among obese and nonobese patients were also extracted and calculated. The reviewers cross-checked all extracted data, and any dissent was resolved by discussion until consensus was reached. The Kappa score was 0.89. The selected studies were assessed for quality by the reviewers using the Cochrane-advocated ROB-2 tool for assessing the risk of bias in RCTs [52]. For non-RCTs, the ROBINS-I tool was used for assessing risk of bias by looking into pre-intervention, intervention and post-intervention domains. Studies were evaluated as low risk of bias (≤ one moderate concern in the included domains, comparable), moderate (≤ four moderate concerns, credible but cannot considered comparable); serious risk of bias (at least one serious concern or multiple moderate concerns; with important problems in the design) and critical risk of bias (critical concerns or multiple serious concerns; unreliable) [46].

### Synthesis of results

Studies with methodological homogeneity were pooled together in a meta-analysis using Review Manager 5.4, which was provided by the Cochrane Collaboration. We assessed statistical inconsistency and heterogeneity by examining the forest plots, Tau^2^, and the I^2^ statistics. Significance levels of overall effects were determined by Z test, and forest plots were provided to demonstrate effect sizes and the corresponding 95% CI. Between-study heterogeneity was estimated by I^2^ statistic, which was based on the Cochran Q statistics [47]. Additionally, unlike merely focus on I^2^ > 50% or not, we evaluated clinical heterogeneity to determine the appropriate effect model. Most of the included studies reported cytokine/adipokine levels in biofluids using medians. For studies that reported median and interquartile ranges, we estimated means and standard deviations through the given series estimation formulas through simulation studies provided by Wan X, McGrath, S. et al. [48, 49, 51]. The significance *p* value was set as 0.05.

### Subgroup analysis

Fifty-four subgroup analyses were conducted based on the biomarker category, biofluid type (serum, saliva, and gingival crevicular fluid (GCF)), participant group, and time of follow-up after NSPT. Subgroup meta-analyses were performed using mean differences (MD) and 95% confidence intervals (CI). The synthesis of results instructions was followed for all subgroup analyses, and sensitivity analysis was performed.

## Results

### Study selection

**Fig. 1:**
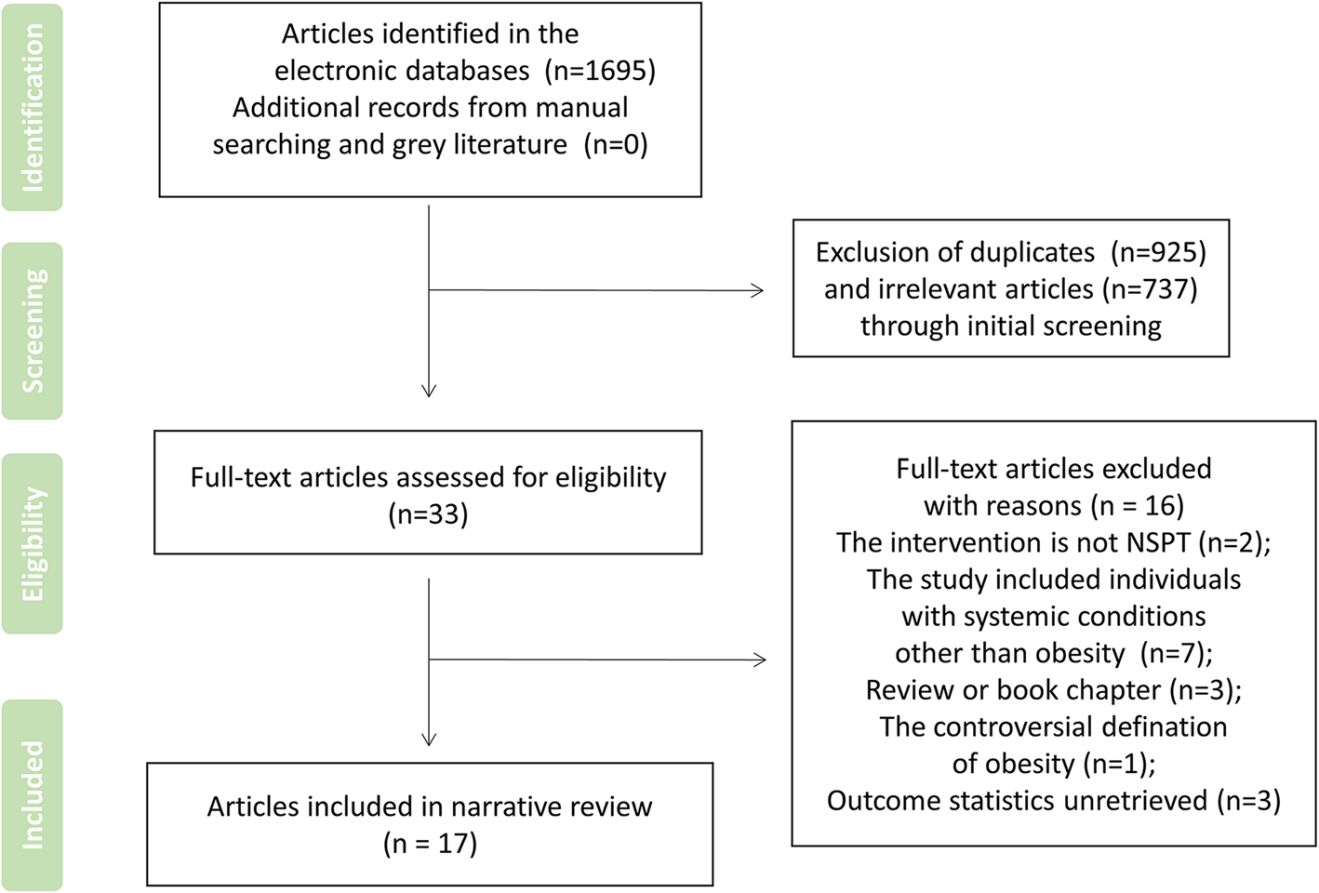
Flowchart presenting the search and the selection phases of the incorporated articles of the systematic review

A total of 1,695 studies were identified in the electronic databases, and no additional record was found from manual searching (duplicated) or the gray literature. Initial screening of the titles and abstracts was followed to remove 925 duplicates and 737 irrelevant studies from the inclusions. Thus, 33 studies were selected for eligibility. After assessment of the full texts of the 33 articles, 16 studies were excluded, and the specific reasons were listed in Table S1 (Supplementary Information File). Seventeen studies were eventually included in the present systematic review, and sixteen were included in meta-analysis. A flowchart presenting all the above-stated phases is displayed in Fig. 1.

### Study characteristics

The characteristics of the 17 incorporated studies are depicted in Table 1. The studies reflected the diversity of ethnicities as they were conducted in different countries; meanwhile, all included studies were published in English. All studies consisted of a group of obese individuals with periodontitis who were treated with NSPT. As for defining parameters of obesity in the included studies, the WHO criteria with or without assistant measures such as body fat, waist-hip ratio, and so on were taken into account. Notably, given by two BMI cut-off point [7, 33] for obesity recommended by WHO as stated in the Obesity and periodontitis criterion, BMI over 27.5 kg/m2 in Asian obesity populations [33] was applied by three included studies in Asian settings [39, 53, 54]. The number of individuals in each study ranged from 40 to 231. The follow-up time of each one ranges from six weeks to two years; the majority is about three months [34–37, 39, 40, 42–45, 54–56] and six months [41, 42, 45, 53] after the NSPT. All studies provided related professional oral hygiene instructions and sufficient periodontal parameters at the follow-up timepoints.

### NSPT and inflammatory-related cytokines/adipokines

Our systematic analysis revealed that among the various inflammatory cytokines, IL-6 and CPR/hs-CRP were the most commonly investigated by researchers. TNF-a, IL-1β, IL-10, and interferon-γ were investigated to a lesser extent. In terms of adipokines, resistin, adiponectin, and leptin were the most widely studied, while RBP4 and vastatin were studied to a lesser extent. The efficacy of NSPT in respect to each of these cytokines and adipokines in different biological fluids (serum, saliva, and GCF) are presented in Tables S2 and S3.

### Evaluation of the methodological quality of the incorporated studies

According to the critical evaluation instruments used (see Supplementary Tables S4 and S5), all randomized and non-randomized studies included in this systematic review and meta-analysis showed a relatively high degree (low or moderate risk of bias) of methodological quality, however, our findings should be interpreted with caution, as most of the published studies were at moderate risk of bias. The basic methodological deficiencies resulted in moderate risk of bias were the missing data due to the dropouts of more than 5% in two studies [43, 45] during follow-up, and failure to prospectively calculate sample size in two studies [37, 41] and the low to moderate quality could be partially attributed to the inevitable presence of confounders (confounders such as unstated smoking issues, degree of pathological condition, etc.).

### Non-surgical periodontal therapy and IL-6

**Fig. 2:**
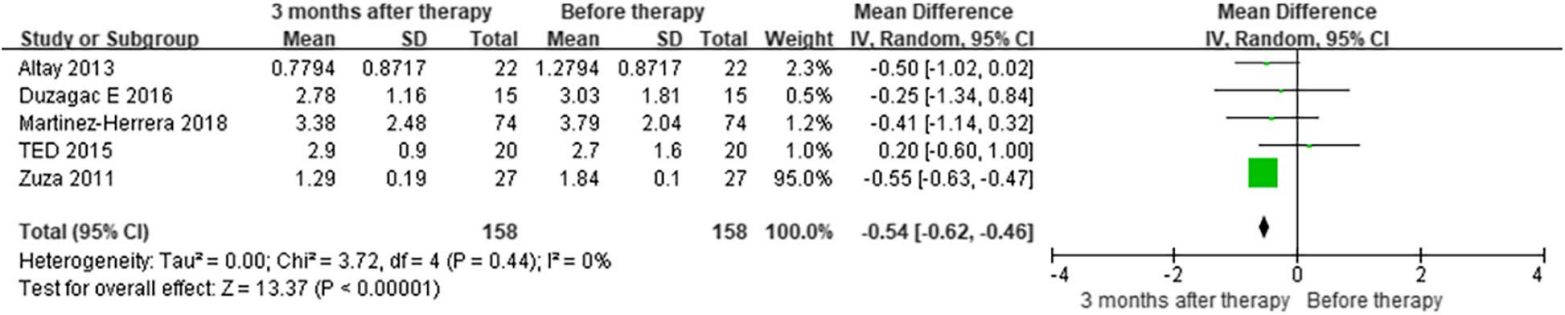
Subgroup analysis comparing serum levels of IL-6 before and three months after non-surgical periodontal therapy in patients with obesity and periodontitis (OP) group

In the subgroup analysis that compared serum levels of IL-6 (pg/mL), five studies [34, 35, 40, 42, 43] showed that the baseline IL-6 levels of people with obesity and periodontitis (OP) were higher than those of normal-weight patients with periodontitis (NP) before treatment (MD = 0.51, CI = 0.09–0.93) (Figure S1). However, there was a reduction in serum levels of IL-6 in OP individuals three months after non-surgical periodontal treatment (NSPT) compared to before treatment (MD = -0.54, CI = -0.62–-0.46) [1–3, 7, 8, 39, 42] (Fig. 2), while there was no difference in serum levels of IL-6 in NP individuals (MD = -0.19, CI = -0.62–0.24) [34, 35, 40, 42, 43] (Figure S2). Furthermore, at the 3-month follow-up, five studies [34, 35, 40, 42, 43] showed that serum IL-6 levels in OP patients did not differ from those in NP patients (MD = 0.42, CI = -0.06–0.90) (MD = 0.42, CI = -0.06–0.90) (Figure S3).

**Fig. 3:**
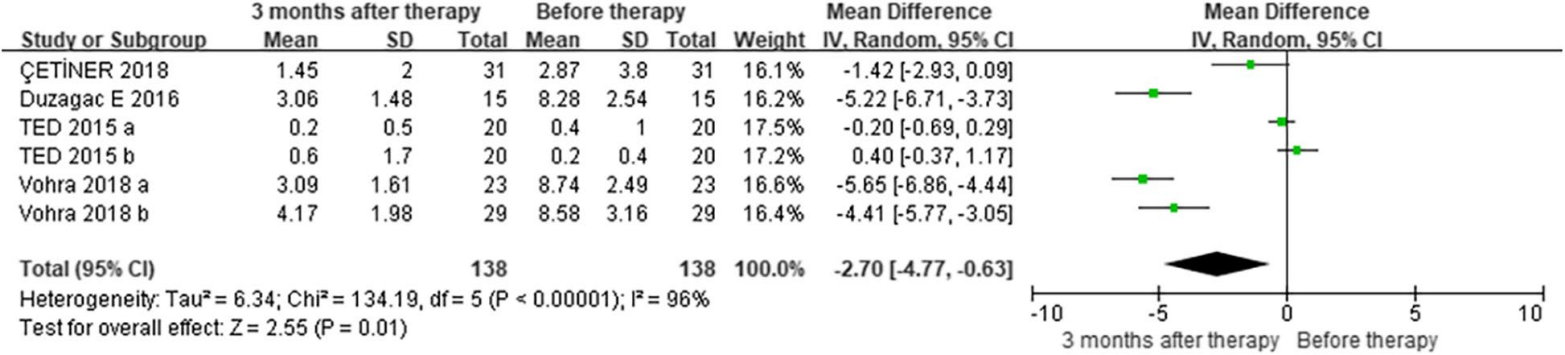
Subgroup analysis comparing GCF levels of IL-6 before and three months after non-surgical periodontal therapy in patients with obesity and periodontitis (OP) groups

**Fig. 4:**
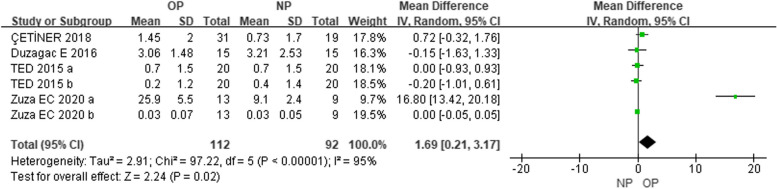
Comparison of distal follow-up time GCF levels of IL-6 after non-surgical periodontal therapy among patients with obesity and periodontitis (OP) and normal-weight patients with periodontitis (NP) groups

In the subgroup analysis that compared GCF levels of IL-6 (pg/mL), three studies [35, 36, 42] showed no difference in IL-6 levels between OP and NP patients at baseline (MD = 0.27, CI = -0.59–1.13) (Figure S4). However, four studies [35, 36, 42, 54] demonstrated a decline in IL-6 levels in OP individuals three months after NSPT compared to before treatment (MD = -2.70, CI = -4.77–-0.63) (Fig. 3), while three studies [35, 36, 42] showed no difference in NP patients (MD = -0.60, CI = -1.38–0.18) (Figure S5). At the 3-month follow-up, three studies showed no difference in GCF IL-6 levels between OP and NP patients (MD = 0.15, CI = -0.32–0.62) [35, 36, 42] (Figure S6). With regard to the long-term effects of NSPT, three studies indicated no difference in IL-6 levels in OP patients (MD = -1.48, CI = -3.37–0.41) [35, 36, 42] (Figure S7) or NP patients (MD = -0.54, CI = -1.54–0.46) [35, 36, 42] (Figure S8) before and after therapy. Overall, four studies [35, 36, 42, 57] reported that the GCF IL-6 levels of OP patients were higher than those of normal-weight periodontitis patients in the longest follow-up time (MD = 1.69, CI = 0.21–3.17) (Fig. 4).

### NSPT and TNF-a

**Fig. 5:**
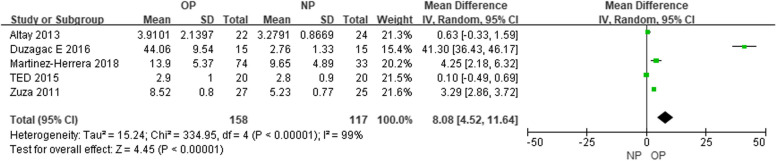
Comparison of follow-up serum levels of TNF-a three months after non-surgical periodontal therapy among patients with obesity and periodontitis (OP) and normal-weight patients with periodontitis (NP) groups

In the subgroup analysis comparing serum levels of TNF-a (pg/mL), five included studies [34, 35, 40, 42, 43] suggested that the level of the OP group was higher than that of the NP group at baseline (MD = 10.36, CI = 4.58–16.15) (Figure S9). Besides, six studies [34, 35, 40, 42–44] indicated that there was no difference of serum TNF-a levels among OP patients before and 3 months after the NSPT (MD = -3.69, CI = -10.29–2.92) (Figure S10), also, five studies [34, 35, 40, 42, 43] indicated there was no difference in NP patients (MD = -3.16, CI = -8.79–2.48) (Figure S11). By the end of 3-month follow-up, five studies [34, 35, 40, 42, 43] suggested the serum TNF-a level of the OP group was still higher than that in NP group (MD = 8.08, CI = 4.52–11.64) (Fig. 5).

**Fig. 6:**
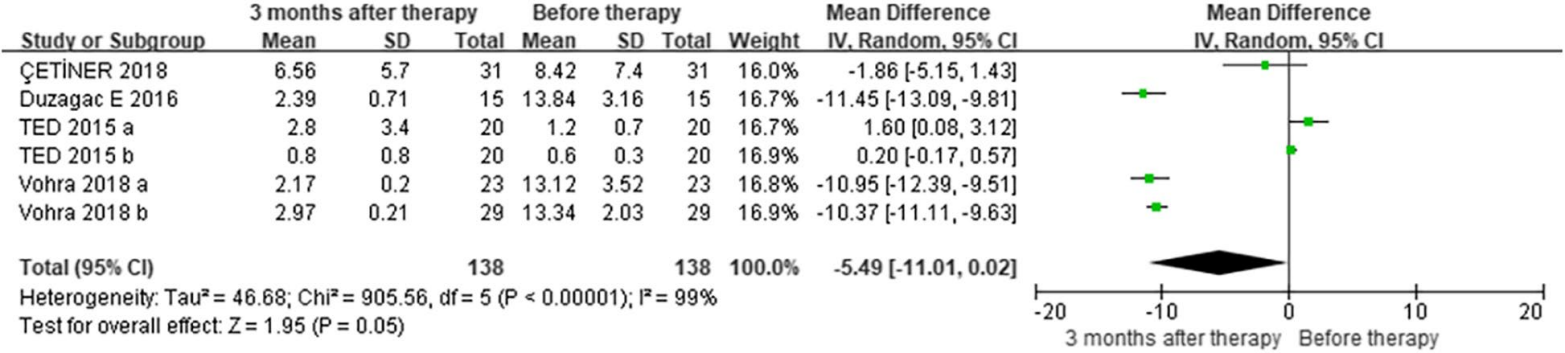
Subgroup analysis comparing GCF levels of TNF-a before and three months after non-surgical periodontal therapy in patients with obesity and periodontitis (OP) groups

As for the subgroup analysis comparing GCF levels of TNF-a (pg/mL), three included studies [35, 36, 42] suggested that the level of the OP group was higher than that in the NP group at baseline (MD = 0.38, CI = 0.17 – 0.59) (Figure S12). Four studies [35, 36, 42, 54] indicated that, for the OP group (MD = -5.49, CI = -11.01 – 0.02) (Fig. 6), or for the NP group (MD = -1.43, CI = -2.84 – 0.03) [35, 36, 42] (Figure S13), the GCF TNF-a level tended to be higher before the NSPT than at the 3-month follow-up. Meanwhile, three studies [35, 36, 42] suggested that there was no difference in GCF TNF-a level between the OP and NP groups at the 3-month follow-up (MD = 0.25, CI = -0.70 – 1.21) (Figure S14). Concerning the distal time effect, four studies [35, 36, 42, 54] indicated that there was hardly any difference before and after the longest follow-up intervals for either the OP group (MD = -5.13, CI = -10.86 – 0.59) (Figure S15) or the NP group (suggested by three studies [35, 36, 42]) (MD = -1.49, CI = -3.22 – 0.25) (Figure S16) individually. Moreover, four studies [35, 36, 42, 57] indicated that the GCF TNF-a level tended to be higher before the NSPT, while actually showing no difference compared to the longest follow-up time points (MD = 1.16, CI = -0.01 – 2.33) (Figure S17).

### NSPT and CRP/hs-CRP

As for the subgroup analysis comparing serum levels of CRP/hs-CRP (mg/L), six studies [35, 37, 40, 41, 43, 58] suggested that the level of OP group was higher than that in NP group at baseline (MD = 5.41, CI = 2.63 – 8.19) (Figure S18). In contrast, four studies [35, 37, 40, 43] suggested that there was no obvious reduction 3 months after the NSPT among OP patients (MD = -2.93, CI = -6.74 – 0.61) (Figure S19) and neither in NP group (MD = -1.39, CI = -3.57 – 0.79) [35, 37, 40, 43] (Figure S20). Further, these same four studies [35, 37, 40, 43] demonstrated that the level of OP individuals was no significant difference as compared with NP individuals 3 months after therapy in serum CRP/hs-CRP level (MD = 5.87, CI = -0.61 – 12.35) (Figure S21).

### NSPT and resistin

In the subgroup analysis comparing salivary levels of resistin (ng/mL), merely two included studies [39, 53] suggested that there was no difference before and after the NSPT within the OP group (MD = -4.60, CI = -12.40–3.20) (Figure S22).

In the subgroup analysis comparing GCF levels of resistin (ng/mL), two included studies [29, 42] indicated that the baseline level of OP individuals was higher than that of NP patients (MD = 2.99, CI = 0.76–5.23) (Figure S23). Moreover, the same two studies [29, 42] suggested the GCF resistin level of OP individuals showed no difference before and after the NSPT (MD = 1.45, CI = -5.60 – 8.50) (Figure S24) as well as that among NP patients (MD = -0.71, CI = -8.14 – 6.73) [29, 42] (Figure S25) with the longest follow-up timepoints. Overall, at the end of the longest follow-up, the GCF resistance level of OP individuals were much higher than that of NP patients (MD = 5.36, CI = 4.18–5.91) [29, 42] (Figure S26).

In the subgroup analysis comparing serum levels of resistin (ng/mL), three included studies [29, 42, 55] indicated that the baseline level of OP individuals was higher than that of NP patients (MD = 4.98, CI = 0.26–9.71) (Figure S27). Nevertheless, no difference was discovered among the OP group before and after the therapy (MD = 1.03, CI = -0.70 – 2.76) [29, 42, 55] (Figure S28), nor among the NP groups with the longest follow-up (MD = -0.25, CI = -3.89–3.38) [29, 42, 55] (Figure S29). At the end of the longest follow-up, the serum resistin level of OP individuals were higher than that of NP patients (MD = 6.30, CI = 0.54–12.05) [29, 42, 55] (Figure S30).

### NSPT and adiponectin

In the subgroup analysis comparing serum levels of adiponectin (ng/mL), only two included studies [42, 45] suggested that the level of the OP group was not different from that in the NP group at baseline (MD = 3.72, CI = -12.39–19.83) (Figure S31). Moreover, three studies [35, 42, 45] suggested that there was no difference before and three months after the NSPT among OP patients (MD = 0.76, CI = -1.96–3.47) (Figure S32), and the same was observed among NP groups (MD = 2.28, CI = -5.57–10.13) [35, 42, 45] (Figure S33). Overall, three studies [35, 42, 45] concluded that there was no difference in serum adiponectin level between OP and NP individuals three months after the NSPT (MD = 0.63, CI = -10.13 – 11.38) (Figure S34). Two studies [42, 45] indicated that there was no difference before and six months after the NSPT among OP patients (MD = -5.48, CI = -22.16–11.21) (Figure S35), and the same was observed in NP [42, 45] groups (MD = -3.83, CI = -18.92–11.26) (Figure S36). Moreover, there was no difference in serum adiponectin level between OP and NP individuals six months after the NSPT (MD = 1.76, CI = -11.84–15.36) [42, 45] (Figure S37).

**Fig. 7:**
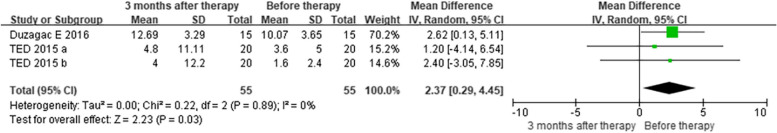
Subgroup analysis comparing GCF levels of adiponectin before and three months after non-surgical periodontal therapy in normal-weight patients with periodontitis (NP) groups

In the subgroup analysis comparing GCF levels of adiponectin (mg/L), only two included studies [35, 42] suggested that the levels of the OP group were not different from those in the NP group at baseline (MD = -0.18, CI = -1.66–1.29) (Figure S38). Moreover, two studies [35, 42] suggested that there was no difference before and three months after the NSPT among OP patients (MD = 1.06, CI = -1.58–3.70) (Figure S39). However, there was an increase in GCF levels of adiponectin three months after the NSPT among NP groups (MD = 2.37, CI = 0.29–4.45) [35, 42] (Fig. 7). Furthermore, there was no difference in GCF levels of adiponectin between the OP and NP individuals three months after the NSPT (MD = -1.15, CI = -3.90–1.60) [35, 42] (Figure S40).

### NSPT and leptin

**Fig. 8:**
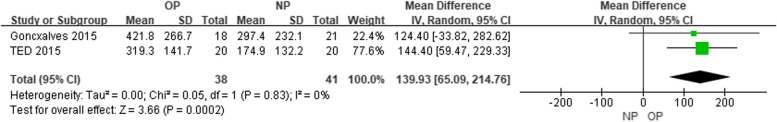
Subgroup analysis comparing 6 months follow-up serum levels of leptin after non-surgical periodontal therapy among patients with obesity and periodontitis (OP) and normal-weight patients with periodontitis (NP) groups

For the subgroup analysis comparing serum levels of leptin (pg/mL), three studies [40, 42, 45] suggested that there was no difference in the level of the OP group compared to that of the NP group at baseline (MD = 126.57, CI = -35.67 – 288.82) (Figure S41). Furthermore, there was no difference before and three months after the NSPT among OP patients (MD = -4.22, CI = -20.92–12.48) [40, 42, 45] (Figure S42) and among NP patients (MD = -3.44, CI = -10.84 – 3.96) [40, 42, 45] (Figure S43). Overall, three studies [40, 42, 45] suggested that there was no difference of serum leptin level between the OP group and the NP group at the 3-month follow-up (MD = 117.94, CI = -32.81–268.70) (Figure S44). Besides, two studies [42, 45] suggested there was no difference before and six months after the NSPT among OP patients (MD = -80.21, CI = -218.17–57.75) (Figure S45) and among NP patients (MD = -8.19, CI = -84.74–68.37) [42, 45] (Figure S46). Overall, two studies [42, 45] suggested that the serum leptin level of the OP group was higher than that of the NP group at the 6-month follow-up (MD = 139.93, CI = 65.09–214.76) (Fig. 8).

### NSPT and RBP4

**Fig. 9:**
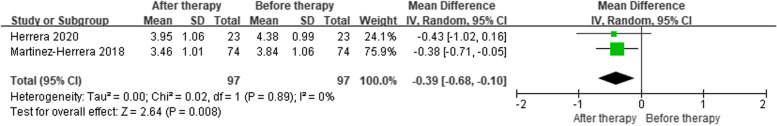
Subgroup analysis comparing serum levels of RBP4 before and 3 months after non-surgical periodontal therapy in patients with obesity and periodontitis (OP) groups

In the subgroup analysis comparing serum levels of RBP4 (mg/dL), only two studies [43, 44] were incorporated, suggesting that the serum RBP4 level of the OP group was higher before the therapy compared with the level at the 3-month follow-up (MD = -0.39, CI = -0.68–0.10) (Fig. 9). Given the clinical heterogeneity of the incorporated studies in the present meta-analysis, the random-effects model was applied in all subgroup analyses. The results of the subgroup analyses are tabulated in Table S3 (Supplementary Information File).

## Discussion

Although the underlying mechanisms of the association between obesity and periodontitis, as well as the interactions between NSPT and both conditions, remain to be fully clarified, research has confirmed the essential role of pro-inflammatory and anti-inflammatory mediators in the development of each condition and their interaction. The link between periodontitis and obesity is dominated by pro-inflammatory factors that exacerbate the severity of both conditions [59, 60]. A meta-analysis has shown that obesity can alter serum levels of pro-inflammatory mediators (i.e., IL-6, CRP, TNF-a, resistin and leptin) in patients with periodontitis, while periodontitis can alter the levels of these mediators in patients with obesity, aggravating the inflammatory profile [61].

Previous studies have primarily focused on observational studies (basically cross-sectional studies) without the introduction of intervention like NSPT, thus unable to assess the changes in levels of relevant mediators after treatment through intervention measures. With the implementation of NSPT, there is bound to be a redistribution of pro-inflammatory and anti-inflammatory mediators, such as a decrease in typical pro-inflammatory mediators like IL-6 and an increase in anti-inflammatory mediators like adiponectin, which is the main focus of our current study. We have analyzed the levels of these mediators in different biological fluids, including whole saliva, GCF, and serum. Saliva and GCF are known to carry local inflammatory mediators. GCF, in particular, is an oral fluid/ exudate that resides in close proximity to gingival tissues and contains various biomarkers and products derived from both hosts and bacteria [62]. Saliva and GCF are reliable tools widely used to detect even small changes during disease processes. They can be collected non-invasively to provide a more precise reflection of the condition of periodontitis. However, unlike whole saliva, which can represent the systemic conditions in general, GCF seems to be more specific. Samples of GCF taken from deep or shallow sites of the periodontal pocket represent different pathological conditions [63]. Meanwhile, serum can reflect the course of multiple diseases more comprehensively, serving as an indicator of the whole system [64].

Interleukin-6, a pro-inflammatory mediator, is known to regulate the host response to both periodontitis bacterial infection and obesity-related disorders [65, 66]. The results of a meta-analysis suggest that individuals with periodontitis and obesity have higher baseline serum levels of IL-6. However, after NSPT, the reduction of IL-6 levels is statistically significant in patients with obesity in a 3-month follow-up period, while no such impact is observed in normal-weight individuals with periodontitis. Thus, IL-6 levels after a 3-month follow-up interval may be an ideal clinical serum bioanalysis to detect the actual effect of NSPT in obesity patients.

Furthermore, the reduction of IL-6 levels in GCF after NSPT is also statistically significant in patients with obesity in a 3-month follow-up period. However, in follow-up periods longer than 3 months but less than 12 months, the impact of NSPT starts to diminish, as the IL-6 levels in GCF gradually increase in obese individuals, resulting in higher levels than those observed in non-obesity groups. These findings suggest that more frequent follow-up assessments are necessary to detect patients' conditions in a timely manner and initiate supplement treatment measures to improve prognosis, considering the time effect of the efficacy of NSPT.

TNF-α, as a pro-inflammatory mediator, has been shown to have high serum levels in individuals with periodontitis, indicating its involvement in the pathologic process of periodontitis. Moreover, TNF-α has been reported to be associated with several diseases, including diabetes, cardiovascular disease, cancer, and metabolic disorders [67]. Notably, TNF-α levels are correlated with the degree of adiposity and the associated insulin resistance [9]. According to the quantitative analysis, the serum level of TNF-α did not change three months after NSPT. Furthermore, both the baseline and three-month follow-up showed higher levels in obesity participants than in normal-weight participants, indicating that the positive effect of treatment on TNF-α serum levels was offset. Additionally, NSPT did not have an impact on TNF-α levels in GCF for either obese or non-obese participants, both at the three-month and distal timepoints. Interestingly, obesity participants presented with higher baseline GCF levels of TNF-α than non-obesity participants.

Elevated plasma levels of CRP, or hs-CRP, have been reported to be linked to obesity and insulin resistance [68, 69], with the latter being implicated in the association between obesity and periodontitis [23, 70]. The meta-analysis results suggest that NSPT may be more effective in reducing overall serum levels of CRP/hs-CRP at the 3-month follow-up, as there was no significant difference between individuals with and without obesity. However, at baseline, serum levels of CRP/hs-CRP were significantly higher in obesity patients than in those without obesity. Despite this, there was no apparent reduction in systemic inflammation in either obesity or non-obese patients as measured by serum levels of CRP/hs-CRP at the 3-month follow-up. Therefore, a 3-month follow-up interval may not be sufficient to support the actual effect of NSPT, and more timepoints need to be accumulated for stronger evidence.

Intriguingly, resistin, a member of the adipokine family, is also a pro-inflammatory mediator that plays a critical role in the development of obesity, insulin resistance, and related comorbidities [71]. Previous studies have shown that resistin levels increase with the severity of periodontal disease and decrease after periodontal therapy [72]. However, the results of quantitative research suggest that NSPT may not have a positive effect on either the GCF or serum levels of resistin in both obese and non-obese individuals at the distal timepoints. Moreover, based on the GCF and serum levels of resistin at baseline and the distal follow-up, it was found that obese patients had a higher expression of resistin, indicating an obesity-dominant higher level of pro-inflammatory expression.

Adiponectin, an anti-inflammatory adipokine, and has been shown to be a regulator of macrophage polarization to assist in reducing inflammation [73]. Besides, adiponectin and its agonists are promising candidates for the treatment of periodontitis due to emerging evidence of the link between adiponectin and periodontitis [74]. Based on the results of our systematic review, two studies [35, 42] demonstrated that NSPT leads to an increase in adiponectin levels in non-obese individuals. However, the meta-analysis suggests that NSPT may not have an impact on the serum levels of adiponectin at 3- or 6-months post-treatment in either obese or non-obese subjects. The same situation was observed in the GCF level of adiponectin at the 3-month follow-up in obese patients, but a significant increase in the GCF level of adiponectin was found in non-obese patients 3 months after the therapy, indicating that obesity may reduce the effectiveness of the treatment, while non-obese individuals may be more responsive to NSPT for anti-inflammation. There was no difference between obese and non-obese participants in the baseline serum level of adiponectin, as well as at the 3-month/6-month follow-up for serum adiponectin and at the 3-month follow-up for GCF adiponectin. Adiponectin may be a sensitive biomarker only in non-obese patients for a 3-month follow-up after NSPT to evaluate the treatment's effectiveness.

Leptin is one of the most well-known pro-inflammatory adipokines and is also recognized for its role in appetite regulation. Leptin resistance is considered a major risk factor for obesity [75]. Recent research has shown that leptin can promote the progression of periodontitis by inducing pro-inflammatory M1 macrophage skewing through the leptin/NLRP3 signaling pathway [76]. Our quantitative analysis of serum leptin levels found that NSPT did not have a significant impact on either obese or normal weight individuals at 3-month or 6-month intervals. Additionally, there were no differences in serum leptin levels between the two groups at baseline or at the 3-month follow-up. However, at the 6-month follow-up, serum leptin levels were significantly higher in the obese group compared to the non-obese group. These findings suggest that serum leptin analysis at either 3-month or 6-month intervals following NSPT may not be an accurate marker for treatment effectiveness.

RBP4, an adipokine related to obesity and insulin resistance [50], has been proposed as a potential biomarker of inflammatory activity in obesity and chronic periodontitis [77]. However, due to the limited number of included studies, a subgroup analysis of RBP4 in serum levels was only conducted in obese individuals. The results suggest that NSPT contributed to a decrease in the serum levels of RBP4 at 3 months post-treatment. This decrease in RBP4 levels may be indicative of a reduction in inflammatory activity in obese individuals.

Despite the above-mentioned biomarkers, MMP-8 is another essential mediator in the systemic subclinical inflammatory response in obesity; moreover, previous research has shown that some functional polymorphisms in the MMP genes are associated with the risk of periodontal disease [78, 79]. Meanwhile, many oxidative stress-related metabolites, such as malondialdehyde and 8-hydroxy-deoxyguanosine in GCF, were the most consistently associated with periodontitis [80]. Besides, patients with chronic periodontitis displayed higher levels of vaspin whether with obesity or not, however, the level of vaspin declined after the NSPT. These findings may provide other ideal diagnostic and prognostic indicators of CP for better therapeutic outcome [81].

The effect of obesity, specifically the adipose tissue, on the expression of pro-inflammatory cytokines may limit the effectiveness of NSPT in reducing their levels. Adipose tissue is an endocrine organ that releases various pro-inflammatory cytokines, which may increase the threshold of the NSPT's effect, making it difficult for the gingiva and periodontal bone to relieve the inflammatory state [82]. The relevant indicators suitable for the comorbidity model of obesity and periodontitis have been screened out from a number of pro-inflammatory and anti-inflammatory cytokines, and the results are considerable. However, due to the limitations of current research data, a longitudinal comparison of given changing levels (*Δ*values for variation for individual before and after the NSPT) of indicators between obese and non-obese subjects cannot be effectively carried out. It is widely known that periodontitis is a bacterial infectious disease, and many cytokines/adipokines related to inflammation regulate the pathophysiology of periodontitis through interactions between tissue cells and immune cells.

Additionally, the regulatory mechanisms involved in the associations between periodontitis and obesity remain to be fully elucidated. It is important to control for confounding factors by excluding individuals with other metabolic or systemic diseases to minimize selection bias, as obesity is often comorbid with other conditions. Furthermore, follow-up intervals should be standardized and the observation period prolonged to evaluate long-term efficacy. As new adipokines, such as vaspin and chemerin, continue to emerge, more research is needed to establish their relationship with periodontitis and obesity. Inevitably, with the only permission of English-published article, publish bias cannot be avoid and our findings should be interpreted with caution, as most of the published studies were at moderate risk of bias and in several meta-analyses the heterogeneity is very high, basically because of the clinical heterogeneity. These limitations present opportunities for future research in this field.

## Conclusions

NSPT can impact the levels of specific pro-inflammatory and anti-inflammatory mediators in biological fluids, both in obese and non-obese individuals, as well as between the two groups. Specifically, NSPT can lead to a decrease in serum and GCF levels of IL-6 and RBP4 in obese individuals after 3 months and an increase in GCF adiponectin levels in normal-weight individuals after 3 months. Besides, for patients with periodontitis, there is no sufficient evidence to prove that obese patients have a statistically significant decrease in the levels of other cytokines compared to patients with normal weight. Our findings implied the potential ideal follow-up intervals and sensitive biomarkers for clinical bioanalysis in personalized decision-making of effect of NSPT due to patients’ BMI value. These findings suggest that the 3-month follow-up interval and the aforementioned biomarkers could be valuable for clinical bioanalysis as an auxiliary chairside tool to evaluate the efficacy of NSPT.

### Supplementary Information

**Additional file 1: Table S1.** Inclusion and exclusion criteria and studies excluded after full-text analysis and related reasons. **Table S2.** The detailed effect of NSPT in respect to inflammatory cytokines and adipokines. **Table S3.** Synthesis of results in subgroup analyses. **Table S4.** Risk of bias assessment of included studies according to the ROBINS-I tool. **Table S5.** Risk of bias assessment of the 4 included RCTs with the ROB-2 tool. **Figure S1.** Subgroup analysis comparing baseline serum levels of IL-6 before non-surgical periodontal therapy within patients with obesity and periodontitis (OP) and normal-weight patients with periodontitis (NP) groups. **Figure S2.** Subgroup analysis comparing serum levels of IL-6 before and three months after non-surgical periodontal therapy in normal-weight patients with periodontitis (NP) group. **Figure S3.** Comparison of 3-month follow-up of serum levels of IL-6 after non-surgical periodontal therapy within patients with obesity and periodontitis (OP) and normal-weight patients with periodontitis (NP) groups. **Figure S4.** Subgroup analysis comparing baseline GCF levels of IL-6 before non-surgical periodontal therapy within patients with obesity and periodontitis (OP) and normal-weight patients with periodontitis (NP) groups. **Figure S5.** Subgroup analysis comparing GCF levels of IL-6 before and three months after non-surgical periodontal therapy in normal-weight patients with periodontitis (NP) groups. **Figure S6.** Comparison of 3-month follow-ups of GCF levels of IL-6 after non-surgical periodontal therapy for patients with obesity and periodontitis (OP) and normal-weight patients with periodontitis (NP) groups. **Figure S7.** Subgroup analysis comparing GCF levels of IL-6 before and distal follow-up time after non-surgical periodontal therapy in patients with obesity and periodontitis (OP) groups. **Figure S8.** Subgroup analysis comparing GCF levels of IL-6 before and distal follow-up time after non-surgical periodontal therapy in normal-weight patients with periodontitis (NP) groups. **Figure S9.** Subgroup analysis comparing baseline serum levels of TNF-a before non-surgical periodontal therapy within patients with obesity and periodontitis (OP) and normal-weight patients with periodontitis (NP) groups. **Figure S10.** Subgroup analysis comparing serum levels of TNF-a before and three months after non-surgical periodontal therapy in patients with obesity and periodontitis (OP) groups. **Figure S11.** Subgroup analysis comparing serum levels of TNF-a before and three months after non-surgical periodontal therapy in normal-weight patients with periodontitis (NP) groups. **Figure S12.** Subgroup analysis comparing baseline GCF levels of TNF-a before non-surgical periodontal therapy within patients with obesity and periodontitis (OP) and normal-weight patients with periodontitis (NP) groups. **Figure S13.** Subgroup analysis comparing GCF levels of TNF-a before and three months after non-surgical periodontal therapy in normal-weight patients with periodontitis (NP) groups. **Figure S14.** Comparison of three-month follow-ups of GCF levels of TNF-a after non-surgical periodontal therapy within patients with obesity and periodontitis (OP) and normal-weight patients with periodontitis (NP) groups. **Figure S15.** Subgroup analysis comparing GCF levels of TNF-a before and distal follow-up time after non-surgical periodontal therapy in patients with obesity and periodontitis (OP) groups. **Figure S16.** Subgroup analysis comparing GCF levels of TNF-a before and distal follow-up time after non-surgical periodontal therapy in normal-weight patients with periodontitis (NP) groups. **Figure S17.** Subgroup analysis comparing distal follow-up time GCF levels of TNF-a after non-surgical periodontal therapy within patients with obesity and periodontitis (OP) and normal-weight patients with periodontitis (NP) groups. **Figure S18.** Subgroup analysis comparing baseline serum levels of CRP/hs-CRP before non-surgical periodontal therapy within patients with obesity and periodontitis (OP) and normal-weight patients with periodontitis (NP) groups. **Figure S19.** Subgroup analysis comparing serum levels of CRP/hs-CRP before and 3 months after non-surgical periodontal therapy in patients with obesity and periodontitis (OP) groups. **Figure S20.** Subgroup analysis comparing serum levels of CRP/hs-CRP before and 3 months after non-surgical periodontal therapy in normal-weight patients with periodontitis (NP) groups. **Figure S21.** Subgroup analysis comparing follow-up serum levels of CRP/hs-CRP 3 months after non-surgical periodontal therapy within patients with obesity and periodontitis (OP) and normal-weight patients with periodontitis (NP) groups. **Figure S22.** Subgroup analysis comparing salivary levels of resistin before and distal time after non-surgical periodontal therapy in patients with obesity and periodontitis (OP) groups. **Figure S23.** Subgroup analysis comparing baseline GCF levels of resistin before non-surgical periodontal therapy within patients with obesity and periodontitis (OP) and normal-weight patients with periodontitis (NP) groups. **Figure S24.** Subgroup analysis comparing GCF levels of resistin before and distal time after non-surgical periodontal therapy in patients with obesity and periodontitis (OP) groups. **Figure S25.** Subgroup analysis comparing GCF levels of resistin before and distal time after non-surgical periodontal therapy in normal-weight patients with periodontitis (NP) groups. **Figure S26.** The comparing distal follow-up time GCF levels of resistin after non-surgical periodontal therapy within patients with obesity and periodontitis (OP) and normal-weight patients with periodontitis (NP) groups. **Figure S27.** Subgroup analysis comparing baseline serum levels of resistin before non-surgical periodontal therapy within patients with obesity and periodontitis (OP) and normal-weight patients with periodontitis (NP) groups. **Figure S28.** Subgroup analysis comparing serum levels of resistin before and distal time after non-surgical periodontal therapy in patients with obesity and periodontitis (OP) groups. **Figure S29.** Subgroup analysis comparing serum levels of resistin before and distal time after non-surgical periodontal therapy in normal-weight patients with periodontitis (NP) groups. **Figure S30.** Comparison of distal follow-up time serum levels of resistin after non-surgical periodontal therapy within patients with obesity and periodontitis (OP) and normal-weight patients with periodontitis (NP) groups. **Figure S31.** Subgroup analysis comparing baseline serum levels of adiponectin before non-surgical periodontal therapy within patients with obesity and periodontitis (OP) and normal-weight patients with periodontitis (NP) groups. **Figure S32.** Subgroup analysis comparing serum levels of adiponectin before and three months after non-surgical periodontal therapy in patients with obesity and periodontitis (OP) groups. **Figure S33.** Subgroup analysis comparing serum levels of adiponectin before and three months after non-surgical periodontal therapy in normal-weight patients with periodontitis (NP) groups. **Figure S34.** Subgroup analysis comparing 3 months follow-up serum levels of adiponectin after non-surgical periodontal therapy within patients with obesity and periodontitis (OP) and normal-weight patients with periodontitis (NP) groups. **Figure S35.** Subgroup analysis comparing serum levels of adiponectin before and six months after non-surgical periodontal therapy in patients with obesity and periodontitis (OP) groups. **Figure S36.** Subgroup analysis comparing serum levels of adiponectin before and six months after non-surgical periodontal therapy in normal-weight patients with periodontitis (NP) groups. **Figure S37.** Subgroup analysis comparing 6 months follow-up serum levels of adiponectin after non-surgical periodontal therapy within patients with obesity and periodontitis (OP) and normal-weight patients with periodontitis (NP) groups. **Figure S38.** Subgroup analysis comparing baseline GCF levels of adiponectin before non-surgical periodontal therapy within patients with obesity and periodontitis (OP) and normal-weight patients with periodontitis (NP) groups. **Figure S39.** Subgroup analysis comparing GCF levels of adiponectin before and three months after non-surgical periodontal therapy in patients with obesity and periodontitis (OP) groups. **Figure S40.** Subgroup analysis comparing 3 months follow-up GCF levels of adiponectin after non-surgical periodontal therapy within patients with obesity and periodontitis (OP) and normal-weight patients with periodontitis (NP) groups. **Figure S41.** Subgroup analysis comparing baseline serum levels of leptin before non-surgical periodontal therapy within patients with obesity and periodontitis (OP) and normal-weight patients with periodontitis (NP) groups. **Figure S42.** Subgroup analysis comparing serum levels of leptin before and three months after non-surgical periodontal therapy in patients with obesity and periodontitis (OP) groups. **Figure S43.** Subgroup analysis comparing serum levels of leptin before and three months after non-surgical periodontal therapy in normal-weight patients with periodontitis (NP) groups. **Figure S44.** Subgroup analysis comparing 3 months follow-up serum levels of leptin after non-surgical periodontal therapy within patients with obesity and periodontitis (OP) and normal-weight patients with periodontitis (NP) groups. **Figure S45.** Subgroup analysis comparing serum levels of leptin before and six months after non-surgical periodontal therapy in patients with obesity and periodontitis (OP) groups. **Figure S46.** Subgroup analysis comparing serum levels of leptin before and six months after non-surgical periodontal therapy in normal-weight patients with periodontitis (NP) groups. 

## Data Availability

The datasets used and/or analyzed during the study are available from the corresponding author upon reasonable request.
