# Supplementary material for: Effect of non-surgical periodontal treatment on cytokines/adipocytokines levels among periodontitis patients with or without obesity: a systematic review and meta-analysis
Source: BMC Oral Health. 2023 Oct 5;23:717. doi: 10.1186/s12903-023-03383-3 (PMC10552206; doi:10.1186/s12903-023-03383-3)
Supplement: Supplementary file 1 — Additional file 1: Table S1. Inclusion and exclusion criteria and studies excluded after full-text analysis and related reasons. Table S2. The detailed effect of NSPT in respect to inflammatory cytokines and adipokines. Table S3. Synthesis of results in subgroup analyses. Table S4. Risk of bias assessment of included studies according to the ROBINS-I tool. Table S5. Risk of bias assessment of the 4 included RCTs with the ROB-2 tool. Figure S1. Subgroup analysis comparing baseline serum levels of IL-6 before non-surgical periodontal therapy within patients with obesity and periodontitis (OP) and normal-weight patients with periodontitis (NP) groups. Figure S2. Subgroup analysis comparing serum levels of IL-6 before and three months after non-surgical periodontal therapy in normal-weight patients with periodontitis (NP) group. Figure S3. Comparison of 3-month follow-up of serum levels of IL-6 after non-surgical periodontal therapy within patients with obesity and periodontitis (OP) and normal-weight patients with periodontitis (NP) groups. Figure S4. Subgroup analysis comparing baseline GCF levels of IL-6 before non-surgical periodontal therapy within patients with obesity and periodontitis (OP) and normal-weight patients with periodontitis (NP) groups. Figure S5. Subgroup analysis comparing GCF levels of IL-6 before and three months after non-surgical periodontal therapy in normal-weight patients with periodontitis (NP) groups. Figure S6. Comparison of 3-month follow-ups of GCF levels of IL-6 after non-surgical periodontal therapy for patients with obesity and periodontitis (OP) and normal-weight patients with periodontitis (NP) groups. Figure S7. Subgroup analysis comparing GCF levels of IL-6 before and distal follow-up time after non-surgical periodontal therapy in patients with obesity and periodontitis (OP) groups. Figure S8. Subgroup analysis comparing GCF levels of IL-6 before and distal follow-up time after non-surgical periodontal therapy in normal-weight pat [file 12903_2023_3383_MOESM1_ESM.docx]

**Supplementary file**

Effect of Non-surgical Periodontal Treatment on Cytokines/Adipocytokines Levels Among Periodontitis Patients with or without Obesity: A Systematic Review and Meta-analysis

Yuwei Zhang^123#^, Ru Jia^123#^, Yifei Zhang^123^, Xuefei Sun^12^, Yukun Mei^123^, Rui Zou^12*^, Lin Niu^123*^, Shaojie Dong^123*^

1. Key Laboratory of Shaanxi Province for Craniofacial Precision Medicine Research, College of Stomatology, Xi’an Jiaotong University, Xi’an 710004, Shaanxi Province, China

2. Clinical Research Center of Shaanxi Province for Dental and Maxillofacial Diseases, Xi’an 710004, Shaanxi Province, China

3. Department of Prosthodontics, College of Stomatology, Xi’an Jiaotong University, Xi’an 710004, Shaanxi Province, China

# These authors contributed equally to this work.

* Corresponding authors.

Rui Zou, rainy@ xjtu.edu.cn; Lin Niu, niulin@xjtu.edu.cn; Shaojie Dong, dongshaojie@xjtu.edu.cn.

Table S1 – Inclusion and exclusion criteria and studies excluded after full-text analysis and related reasons

| Inclusion criteria | Exclusion criteria |
| --- | --- |
| Randomized clinical trials  Controlled clinical trials  Reported the given cytokines/ adipocytokines levels  Published in English | Laboratory animal science  Case-control studies  Cross-sectional  Case series  Systematic reviews  Literature reviews  Data published as conference abstracts  Studies without certain MD or CI data  Clinical analysis involving periodontitis individuals with systemic conditions other than obesity  Participants receive treatments more than NSPT |
| Study | Reason for exclusion |
| Park HS, Nam HS, Seo HS, Hwang SJ. Change of periodontal inflammatory indicators through a 4-week weight control intervention including caloric restriction and exercise training in young Koreans: a pilot study. BMC Oral Health. 2015;15(1):109. Published 2015 Sep 18. doi:10.1186/s12903-015-0094-7 | The intervention of the study was a four-week weight control program rather than non-surgical periodontal therapy. |
| Offenbacher S, Beck JD, Moss K, et al. Results from the Periodontitis and Vascular Events (PAVE) Study: a pilot multicentered, randomized, controlled trial to study effects of periodontal therapy in a secondary prevention model of cardiovascular disease. J Periodontol. 2009;80(2):190-201. doi:10.1902/jop.2009.080007 | The study included individuals with cardiovascular diseases. |
| López NJ, Quintero A, Casanova PA, Ibieta CI, Baelum V, López R. Effects of periodontal therapy on systemic markers of inflammation in patients with metabolic syndrome: a controlled clinical trial. J Periodontol. 2012;83(3):267-278. doi:10.1902/jop.2011.110227 | The study included individuals with metabolic syndrome. |
| Skilton MR, Maple-Brown LJ, Kapellas K, et al. The effect of a periodontal intervention on cardiovascular risk markers in Indigenous Australians with periodontal disease: the PerioCardio study. BMC Public Health. 2011;11:729. Published 2011 Sep 26. doi:10.1186/1471-2458-11-729 | The study is a protocol for studies of individuals with cardiovascular diseases. |
| Kapellas K, Mejia G, Bartold PM, et al. Periodontal therapy and glycaemic control among individuals with type 2 diabetes: reflections from the PerioCardio study. Int J Dent Hyg. 2017;15(4):e42-e51. doi:10.1111/idh.12234 | The study included individuals with type 2 diabetes. |
| Balli U, Ongoz Dede F, Bozkurt Dogan S, Gulsoy Z, Sertoglu E. Chemerin and interleukin-6 levels in obese individuals following periodontal treatment. Oral Dis. 2016;22(7):673-680. doi:10.1111/odi.12520 | In this study, the specific statistics of chemerin and interleukin-6 levels could not be retrieved. |
| Varghese T, Prashant MC, Dodani K, Nagpal N, Khare N, Singh V. Resistin and Plasma-reactive Oxygen Metabolite Levels in Obese and Non-obese Individuals with Chronic Periodontitis in Response to Non-surgical Periodontal Therapy. J Contemp Dent Pract. 2018;19(12):1525-1530. Published 2018 Dec 1. | In this study, the specific statistics of resistin levels could not be retrieved. |
| Wanichkittikul N, Laohapand P, Mansa-Nguan C, Thanakun S. Periodontal Treatment Improves Serum Levels of Leptin, Adiponectin, and C-Reactive Protein in Thai Patients with Overweight or Obesity. Int J Dent. 2021;2021:6660097. Published 2021 Feb 2. doi:10.1155/2021/6660097 | In this study, the defination of obesity is controversial. |
| Matern J, Koch R, Petersmann A, et al. Effect of periodontal therapy on adipokine biomarkers in overweight. J Clin Periodontol. 2020;47(7):842-850. doi:10.1111/jcpe.13288 | The study included individuals with overweight. |
| Alkan B, Guzeldemir-Akcakanat E, Odabas-Ozgur B, et al. Effects of exercise on periodontal parameters in obese women. Niger J Clin Pract. 2020;23(10):1345-1355. doi:10.4103/njcp.njcp_627_19 | The intervention of the study was exercise rather than non-surgical periodontal therapy. |
| Sun WL, Chen LL, Zhang SZ, Wu YM, Ren YZ, Qin GM. Inflammatory cytokines, adiponectin, insulin resistance and metabolic control after periodontal intervention in patients with type 2 diabetes and chronic periodontitis. Intern Med. 2011;50(15):1569-1574. doi:10.2169/internalmedicine.50.5166 | The study included individuals with type 2 diabetes. |
| Mainas G, Ide M, Rizzo M, Magan-Fernandez A, Mesa F, Nibali L. Managing the Systemic Impact of Periodontitis. Medicina (Kaunas). 2022;58(5):621. Published 2022 Apr 29. doi:10.3390/medicina58050621 | This article is a review. |
| Deschner J, Eick S, Damanaki A, Nokhbehsaim M. The role of adipokines in periodontal infection and healing. Mol Oral Microbiol. 2014;29(6):258-269. doi:10.1111/omi.12070 | This article is a review. |
| El-Shinnawi U, Soory M. Associations between periodontitis and systemic inflammatory diseases: response to treatment. Recent Pat Endocr Metab Immune Drug Discov. 2013;7(3):169-188. doi:10.2174/18715303113139990040 | This article is a review. |
| Acharya A, Bhavsar N, Jadav B, Parikh H. Cardioprotective effect of periodontal therapy in metabolic syndrome: a pilot study in Indian subjects. Metab Syndr Relat Disord. 2010;8(4):335-341. doi:10.1089/met.2010.0002 | The study included individuals with metabolic syndrome. |
| Balli U, Bozkurt Dogan S, Ongoz Dede F, Sertoglu E, Keles GC. The levels of visceral adipose tissue-derived serpin, omentin-1 and tumor necrosis factor-α in the gingival crevicular fluid of obese patients following periodontal therapy. J Oral Sci. 2016;58(4):465-473. doi:10.2334/josnusd.16-0212 | In this study, the specific statistics of resistin levels could not be retrieved. |

Table S2 The detailed effect of NSPT in respect to inflammatory cytokines and adipokines

| Biomarker | Biofluid | Outcomes | | |
| --- | --- | --- | --- | --- |
|  |  | Obesity | Non-obesity | Between groups |
| IL-6 | Salivary | One study [1] demonstrated that NSPT contributes to the reduction | One study [1] demonstrated that NSPT contributes to the reduction | One study indicated that [1] the decline of the NP is larger |
|  | Serum | Three studies [2-4] demonstrated that NSPT contributes to the reduction;  Two studies [5, 6] demonstrated that NSPT had no impact | Three studies [2-4] demonstrated that NSPT contributes to the reduction;  One study [5] demonstrated that  NSPT contributes to the rise;  One study [6] demonstrated that NSPT had no impact | Two studies indicated that [2, 4] the decline of the NP is larger |
|  | GCF | Three studies [4, 5, 7] demonstrated that NSPT contributes to the reduction;  One study [8] demonstrated that NSPT had no impact | Two studies [4, 5] demonstrated that NSPT contributes to the reduction  One study [8] demonstrated that NSPT had no impact | One study indicated that [4] the decline of the NP is larger;  One study indicated that [5] the decline of the OP is larger |
| TNF-a | Serum | Four studies [2, 3, 5, 6] demonstrated that NSPT contributes to the reduction;  One study [9] demonstrated that NSPT had no impact | Three studies [2, 5, 6] demonstrated that NSPT contributes to the reduction;  One study [3] demonstrated that NSPT had no impact | Three studies indicated that [2, 3, 6] the decline of the OP is larger;  One study indicated that [5] the decline of the NP is larger |
|  | GCF | Two studies [5, 7] demonstrated that  NSPT contributes to the reduction;  Two studies [4, 8] demonstrated that NSPT had no impact | Two studies [4, 5] demonstrated that  NSPT contributes to the reduction;  One study [8] demonstrated that  NSPT had no impact | - |
| CRP/hs-CRP | Serum | Four studies [3, 10-12] demonstrated that NSPT contributes to the reduction;  Three studies [5, 6, 9] demonstrated that NSPT had no impact | Four studies [3, 5, 10, 12] demonstrated that NSPT contributes to the reduction;  Two studies [6, 11] demonstrated that NSPT had no impact | Two studies indicated that [5, 12] the decline of the NP is larger;  Three studies indicated that [3, 10, 11] the decline of the OP is larger |
| IL-1β | Serum | One study [2] demonstrated that  NSPT contributes to the reduction | One study [2] demonstrated that  NSPT contributes to the reduction | One study indicated that [2] the decline of the OP is larger |
| IFN-γ | Serum | One study [2] demonstrated that  NSPT contributes to the reduction | One study [2] demonstrated that  NSPT contributes to the reduction | One study indicated that [2] the decline of the OP is larger |
| IL-10 | Serum | One study [5] demonstrated that NSPT contributes to the reduction | One study [5] demonstrated that NSPT contributes to the rise | - |
|  | GCF | One study [5] demonstrated that NSPT contributes to the rise | One study [5] demonstrated that NSPT contributes to the rise | One study indicated that [5] the rise of the OP is larger |
| Resistin | Salivary | Two studies [1, 13] demonstrated that NSPT contributes to the reduction | One study [1] demonstrated that  NSPT contributes to the reduction | One study indicated that [1] the decline of the NP is larger |
|  | Serum | Two studies [4, 14] demonstrated that NSPT contributes to the rise;  One study [15] demonstrated that  NSPT had no impact | One study [14] demonstrated that NSPT contributes to the rise;  Two studies [4, 15] demonstrated that  NSPT contributes to the reduction | Two studies indicated that [4, 14] there was no significant in the changes between two groups.  One study indicated that [15] the decline of the NP is larger |
|  | GCF | Two studies [4, 15] demonstrated that NSPT contributes to the reduction | One study [4] demonstrated that NSPT contributes to the rise;  One study [15] demonstrated that  NSPT contributes to the reduction | One study indicated that [4] the decline of the OP is larger;  One study indicated that [15] the decline of the NP is larger |
| Adiponectin | Salivary | One study [16] demonstrated that  NSPT contributes to the reduction | One study [16] demonstrated that  NSPT contributes to the reduction | One study indicated that [16] the decline of the NP is larger |
|  | Serum | Three studies [4, 5, 17] demonstrated that NSPT had no impact | One study [4] demonstrated that NSPT contributes to the reduction;  One study [5] demonstrated that NSPT contributes to the rise;  One study [17] demonstrated that NSPT had no impact | One study indicated that [4] the decline of the NP is larger;  One study indicated that [5] the rise of the NP is larger |
|  | GCF | One study [4] demonstrated that NSPT contributes to the reduction;  One study [5] demonstrated that NSPT had no impact | Two studies [4, 5] demonstrated that  NSPT contributes to the rise | One study indicated that [4] the decline of the OP is larger;  One study indicated that [5] there was no significant in the changes between two groups |
| Leptin | Salivary | One study [16] demonstrated that  NSPT contributes to the reduction | One study [16] demonstrated that  NSPT contributes to the reduction | One study indicated that [16] the decline of the NP is larger |
|  | Serum | Two studies [3, 4] demonstrated that  NSPT contributes to the reduction;  One study [17] demonstrated that  NSPT contributes to the rise | One study [4] demonstrated that  NSPT contributes to the reduction;  One study [3] demonstrated that NSPT had no impact;  One study [17] demonstrated that  NSPT contributes to the rise | Two studies indicated that [3, 4] the decline of the OP is larger |
|  | GCF | One study [4] demonstrated that  NSPT contributes to the reduction | One study [4] demonstrated that  NSPT contributes to the reduction | One study indicated that [4] the decline of the NP is larger |
| Visfatin | GCF | One study [8] demonstrated that  NSPT contributes to the reduction | One study [8] demonstrated that  NSPT had no impact | One study indicated that [8] the decline of the OP is larger |
| RBP4 | Serum | Two studies [6, 9] demonstrated that  NSPT contributes to the reduction | One study [6] demonstrated that  NSPT contributes to the reduction | One study indicated that [6] the decline of the OP is larger |

Table S3 - Synthesis of results in subgroup analyses.

| Biomarker | Biofluid | Outcomes | | | |
| --- | --- | --- | --- | --- | --- |
|  |  | Time | Obesity | Non-obesity | Between groups (O-nO) |
| IL-6 | Serum | 3 mon | MD = -0.54, CI = -0.62 – -0.46* | MD = -0.19, CI = -0.62 – 0.24 | Baseline: MD = 0.51, CI = 0.09 – 0.93*  Follow-up: MD = 0.42, CI = -0.06 – 0.90 |
|  | GCF | 3 mon | MD = -2.70, CI = -4.77 – -0.63* | MD = -0.60, CI = -1.38 – 0.18 | Baseline: MD = 0.27, CI = -0.59 – 1.13  Follow-up: MD = 0.15, CI = -0.32 – 0.62 |
|  |  | Distal time | MD = -1.48, CI = -3.37 – 0.41 | MD = -0.54, CI = -1.54 – 0.46 | Baseline: MD = 0.27, CI = -0.59 – 1.13  Follow-up: MD = 1.69, CI = 0.21 – 3.17* |
| TNF-a | Serum | 3 mon | MD = -3.69, CI = -10.29 – 2.92 | MD = -3.16, CI = -8.79 – 2.48 | Baseline: MD = 10.36, CI = 4.58 – 16.15*  Follow-up: MD = 8.08, CI = 4.52 – 11.64* |
|  | GCF | 3 mon | MD = -5.49, CI = -11.01 – 0.02 | MD = -1.43, CI = -2.84 – 0.03 | Baseline: MD = 0.38, CI = 0.17 – 0.59*  Follow-up: MD = 0.25, CI = -0.70 – 1.21 |
|  |  | Distal time | MD = -5.13, CI = -10.86 – 0.59 | MD = -1.49, CI = -3.22 – 0.25 | Baseline: MD = 0.38, CI = 0.17 – 0.59*  Follow-up:MD = 1.16, CI = -0.01 – 2.33 |
| CRP/  hs-CRP | Serum | 3 mon | MD = -2.93, CI = -6.74 – 0.61 | MD = -1.39, CI = -3.57 – 0.79 | Baseline: MD = 5.41, CI = 2.63 – 8.19*  Follow-up: MD = 5.87, CI = -0.61 – 12.35 |
| Resistin | Salivary | Distal time | MD = -4.60, CI = -12.40 – 3.20 | - | Baseline: -  Follow-up: - |
|  | GCF | Distal time | MD = 1.45, CI = -5.60 – 8.50 | MD = -0.71, CI = -8.14 – 6.73 | Baseline: MD = 2.99, CI = 0.76 – 5.23*  Follow-up: MD = 5.36, CI = 4.18 – 5.91* |
|  | Serum | Distal time | MD = 1.03, CI = -0.70 – 2.76 | MD = -0.25, CI = -3.89 – 3.38 | Baseline: MD = 4.98, CI = 0.26 – 9.71*  Follow-up: MD = 6.30, CI =0.54 – 12.05* |
| Adiponectin | Serum | 3 mon | MD = 0.76, CI = -1.96 – 3.47 | MD = 2.28, CI = -5.57 – 10.13 | Baseline: MD = 3.72, CI = -12.39 – 19.83  Follow-up: MD = 0.63, CI = -10.13 – 11.38 |
|  |  | 6 mon | MD = -5.48, CI = -22.16 – 11.21 | MD = -3.83, CI = -18.92 – 11.26 | Baseline: MD = 3.72, CI = -12.39 – 19.83  Follow-up: MD = 1.76, CI = -11.84 – 15.36 |
|  | GCF | 3 mon | MD = 1.06, CI = -1.58 – 3.70 | MD = 2.37, CI = 0.29 – 4.45* | Baseline: MD = -0.18, CI = -1.66 – 1.29  Follow-up: MD = -1.15, CI = -3.90 – 1.60 |
| Leptin | Serum | 3 mon | MD = -4.22, CI = -20.92 – 12.48 | MD = -3.44, CI = -10.84 – 3.96 | Baseline: MD = 126.57, CI = -35.67 – 288.82  Follow-up: MD = 117.94, CI = -32.81 – 268.70 |
|  |  | 6 mon | MD = -80.21, CI = -218.17 – 57.75 | MD = -8.19, CI = -84.74 – 68.37 | Baseline: MD = 126.57, CI = -35.67 – 288.82  Follow-up: MD = 139.93, CI = 65.09 – 214.76* |
| RBP4 | Serum | 3 mon | MD = -0.39, CI = -0.68 – -0.10* | - | Baseline:-  Follow-up:- |

*-There was difference. (p ≥ 0.05)

Table S4 - Risk of bias assessment of included studies according to the ROBINS-I tool.

.

|  | Al-Hamoudi et al. 2017 | Zuza et al.  2011 | TED et al. 2015 | Al-Zahrani et al. 2012 | Tahir  et al.  2020 | Duzagac E et al. 2016 | ÇETİNER et al. 2018 | Suresh et al.  2018 | Zuza et al. 2016 | Martinez-Herrera  et al.  2018 | Goncxalves et al.  2015 | Altay  et al.  2013 | Suvan  et al.  2021 |
| --- | --- | --- | --- | --- | --- | --- | --- | --- | --- | --- | --- | --- | --- |
| Domain 1:  Confounding  factors | Low | Moderate | Low | Moderate | Moderate | Low | Moderate | Moderate | Moderate | Low | Moderate | Moderate | Moderate |
| Domain 2:  Selection of  participants | Low | Low | Low | Low | Low | Low | Low | Low | Moderate | Low | Low | Low | Moderate |
| Domain 3:  Intervention  classification | Low | Low | Low | Low | Low | Low | Low | Low | Low | Low | Low | Low | Low |
| Domain 4:  Deviation from  intervention | Low | Low | Low | Low | Low | Low | Low | Low | Low | Low | Low | Low | Low |
| Domain 5:  Missing data | Low | Low | Low | Low | Low | Low | Low | Low | Low | Moderate | Moderate | Low | Low |
| Domain 6:  Measurement  of outcome | Moderate | Moderate | Moderate | Moderate | Moderate | Moderate | Moderate | Moderate | Moderate | Moderate | Moderate | Moderate | Moderate |
| Domain 7:  Selection of  reported result | Low | Moderate | Low | Moderate | Moderate | Low | Moderate | Low | Moderate | Moderate | Moderate | Moderate | Moderate |
| ROBINS-I  overall score | Low | Low | Low | Moderate | Moderate | Low | Moderate | Moderate | Moderate | Moderate | Moderate | Moderate | Moderate |

† ROBINS-I is a tool which assesses the risk of bias in non-randomized studies of interventions by looking into pre-intervention, intervention and post-intervention domains.

Studies with low risk of bias (no or one moderate concern in the included domains) are comparable to randomized controlled trials. Studies with moderate risk of bias (up to

four moderate concerns in the included domains) can be characterized as credible but cannot considered comparable to a well performed randomized trial. Studies with serious

risk of bias (at least one serious concern or multiple moderate concerns in the included domains) have important problems in the design. Studies with critical risk of bias (critical

concerns or multiple serious concerns in the included domains) are too problematic to provide useful evidence on the intervention effect.

Table S5- Risk of bias assessment of the 4 included RCTs with the ROB-2 tool.

| Study | Randomization  process | Assignment to  intervention | Adhering to  intervention | Missing outcome  data | Measurement of  outcome | Selection of the  reported result | RoB-2  overall score |
| --- | --- | --- | --- | --- | --- | --- | --- |
| Vohra et al. 2018 | Low risk | Low risk | Low risk | Low risk | Low risk | Some concern | Low risk |
| Akram et al. 2017 | Low risk | Low risk | Low risk | Low risk | Low risk | Some concern | Low risk |
| Herrera et al. 2020 | Low risk | Low risk | Low risk | Low risk | Low risk | Some concern | Low risk |
| Abdellatif et al. 2022 | Low risk | Low risk | Low risk | Low risk | Low risk | Some concern | Low risk |


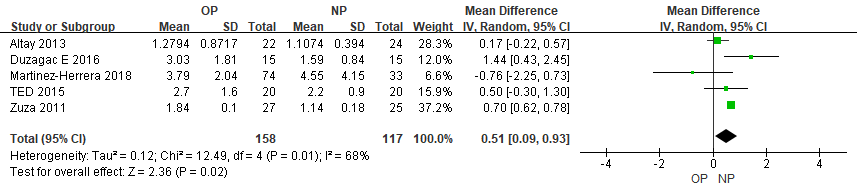


**Figure S1.** Subgroup analysis comparing baseline serum levels of IL-6 before non-surgical periodontal therapy within patients with obesity and periodontitis (OP) and normal-weight patients with periodontitis (NP) groups


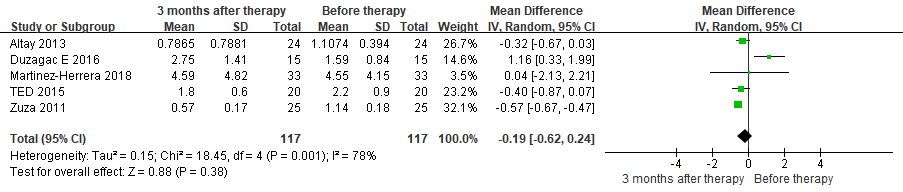


**Figure S2.** Subgroup analysis comparing serum levels of IL-6 before and three months after non-surgical periodontal therapy in normal-weight patients with periodontitis (NP) group.


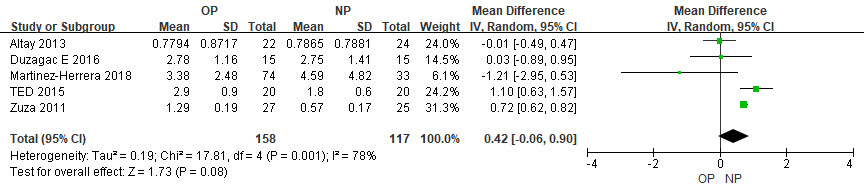


**Figure S3.** Comparison of 3-month follow-up of serum levels of IL-6 after non-surgical periodontal therapy within patients with obesity and periodontitis (OP) and normal-weight patients with periodontitis (NP) groups.


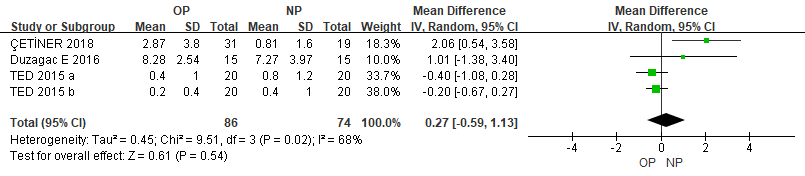


**Figure S4**. Subgroup analysis comparing baseline GCF levels of IL-6 before non-surgical periodontal therapy within patients with obesity and periodontitis (OP) and normal-weight patients with periodontitis (NP) groups.


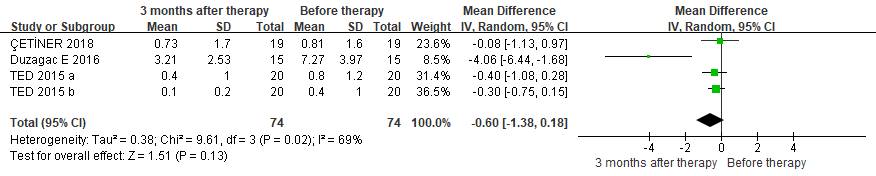


**Figure S5.** Subgroup analysis comparing GCF levels of IL-6 before and three months after non-surgical periodontal therapy in normal-weight patients with periodontitis (NP) groups.


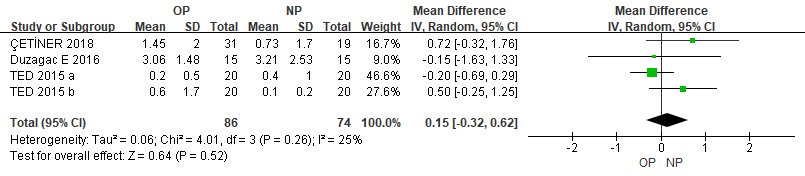


**Figure S6.** Comparison of 3-month follow-ups of GCF levels of IL-6 after non-surgical periodontal therapy for patients with obesity and periodontitis (OP) and normal-weight patients with periodontitis (NP) groups.


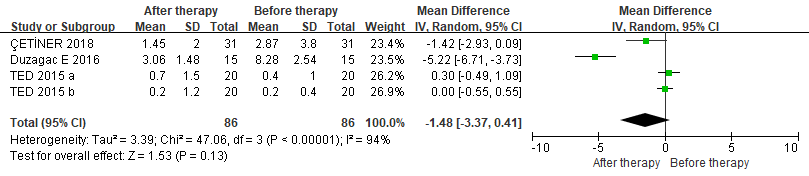


**Figure S7.** Subgroup analysis comparing GCF levels of IL-6 before and distal follow-up time after non-surgical periodontal therapy in patients with obesity and periodontitis (OP) groups.


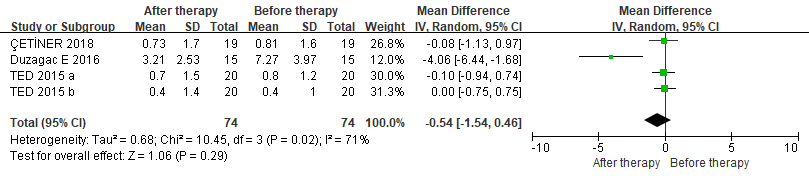


**Figure S8.** Subgroup analysis comparing GCF levels of IL-6 before and distal follow-up time after non-surgical periodontal therapy in normal-weight patients with periodontitis (NP) groups.


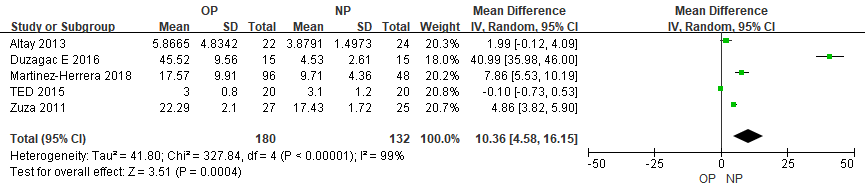


**Figure S9.** Subgroup analysis comparing baseline serum levels of TNF-a before non-surgical periodontal therapy within patients with obesity and periodontitis (OP) and normal-weight patients with periodontitis (NP) groups.


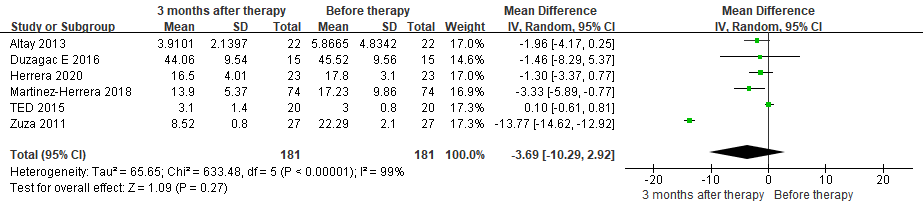


**Figure S10.** Subgroup analysis comparing serum levels of TNF-a before and three months after non-surgical periodontal therapy in patients with obesity and periodontitis (OP) groups.


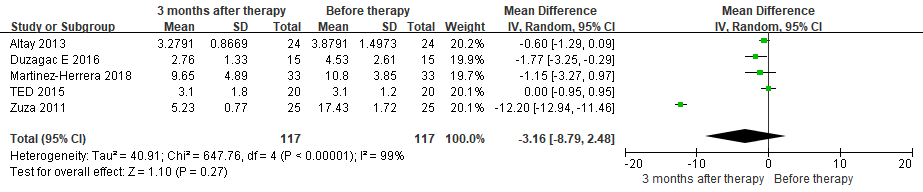


**Figure S11.** Subgroup analysis comparing serum levels of TNF-a before and three months after non-surgical periodontal therapy in normal-weight patients with periodontitis (NP) groups.


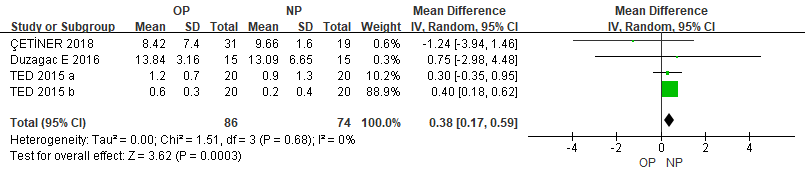


**Figure S12**. Subgroup analysis comparing baseline GCF levels of TNF-a before non-surgical periodontal therapy within patients with obesity and periodontitis (OP) and normal-weight patients with periodontitis (NP) groups.


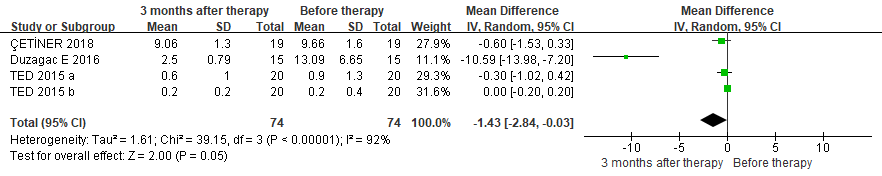


**Figure S13.** Subgroup analysis comparing GCF levels of TNF-a before and three months after non-surgical periodontal therapy in normal-weight patients with periodontitis (NP) groups.


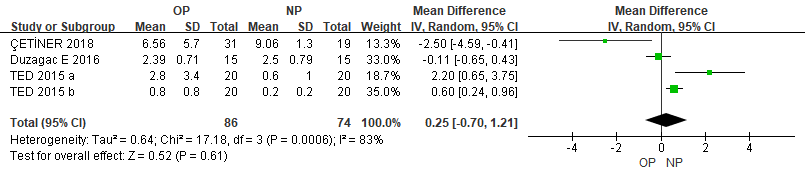


**Figure S14.** Comparison of three-month follow-ups of GCF levels of TNF-a after non-surgical periodontal therapy within patients with obesity and periodontitis (OP) and normal-weight patients with periodontitis (NP) groups.


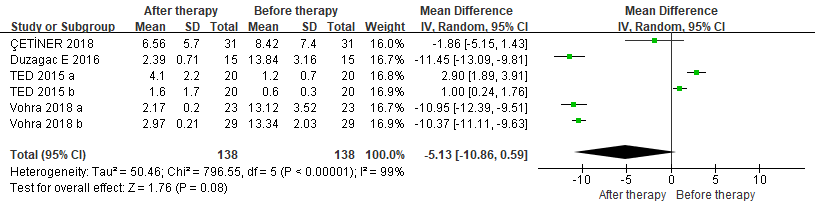


**Figure S15.** Subgroup analysis comparing GCF levels of TNF-a before and distal follow-up time after non-surgical periodontal therapy in patients with obesity and periodontitis (OP) groups.


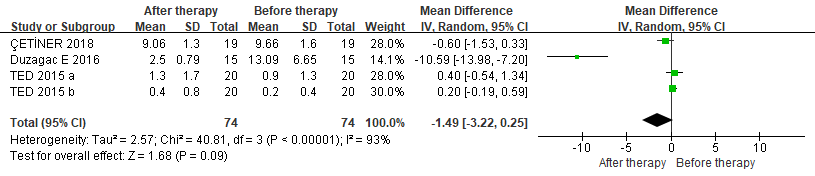


**Figure S16.** Subgroup analysis comparing GCF levels of TNF-a before and distal follow-up time after non-surgical periodontal therapy in normal-weight patients with periodontitis (NP) groups.


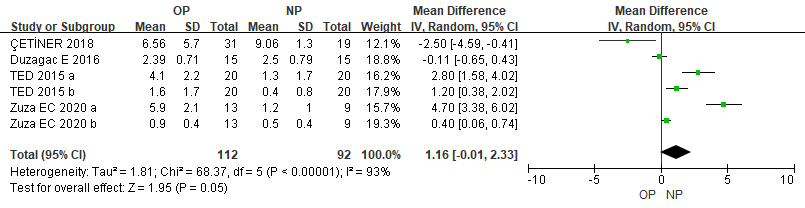


**Figure S17.** Subgroup analysis comparing distal follow-up time GCF levels of TNF-a after non-surgical periodontal therapy within patients with obesity and periodontitis (OP) and normal-weight patients with periodontitis (NP) groups.


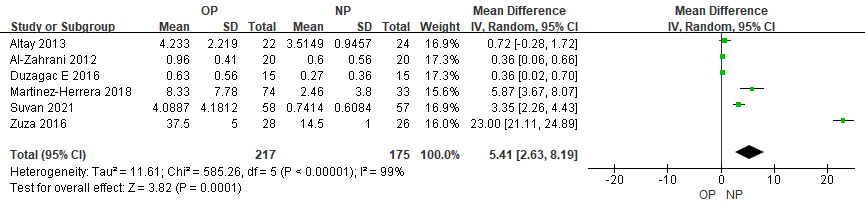


**Figure S18.** Subgroup analysis comparing baseline serum levels of CRP/hs-CRP before non-surgical periodontal therapy within patients with obesity and periodontitis (OP) and normal-weight patients with periodontitis (NP) groups.


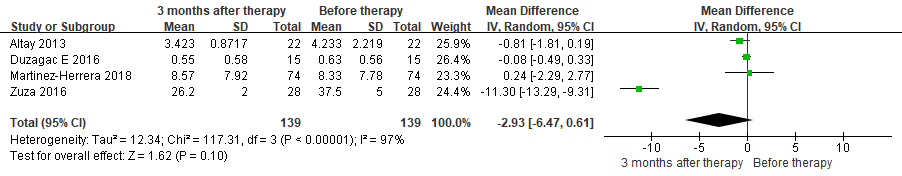


**Figure S19.** Subgroup analysis comparing serum levels of CRP/hs-CRP before and 3 months after non-surgical periodontal therapy in patients with obesity and periodontitis (OP) groups.


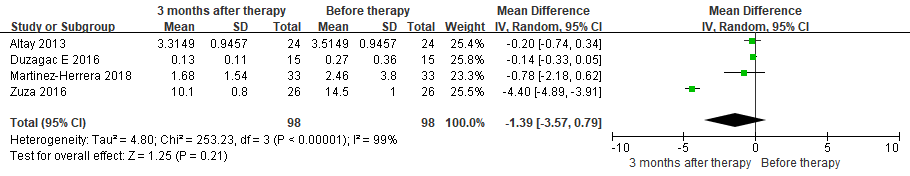


**Figure S20.** Subgroup analysis comparing serum levels of CRP/hs-CRP before and 3 months after non-surgical periodontal therapy in normal-weight patients with periodontitis (NP) groups.


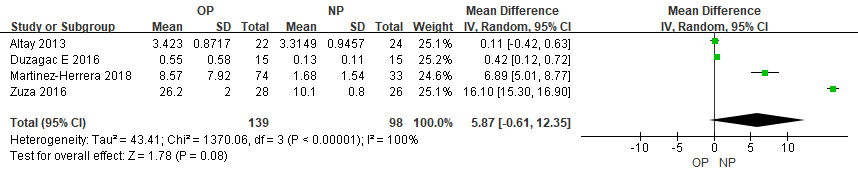


**Figure S21.** Subgroup analysis comparing follow-up serum levels of CRP/hs-CRP 3 months after non-surgical periodontal therapy within patients with obesity and periodontitis (OP) and normal-weight patients with periodontitis (NP) groups.


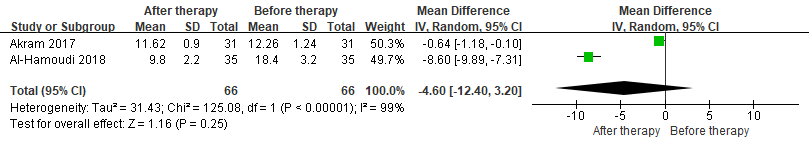


**Figure S22.** Subgroup analysis comparing salivary levels of resistin before and distal time after non-surgical periodontal therapy in patients with obesity and periodontitis (OP) groups


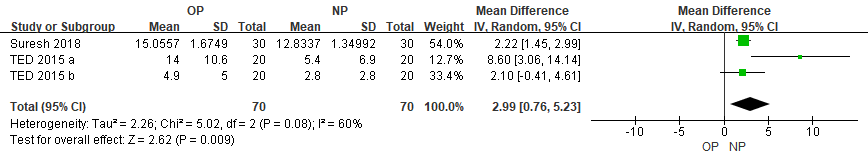


**Figure S23.** Subgroup analysis comparing baseline GCF levels of resistin before non-surgical periodontal therapy within patients with obesity and periodontitis (OP) and normal-weight patients with periodontitis (NP) groups.


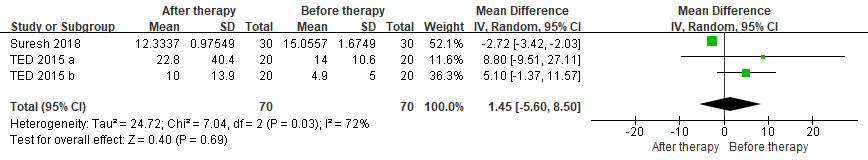


**Figure S24.** Subgroup analysis comparing GCF levels of resistin before and distal time after non-surgical periodontal therapy in patients with obesity and periodontitis (OP) groups.


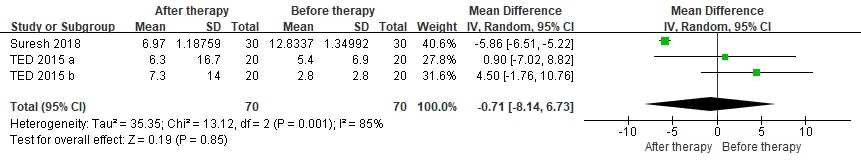


**Figure S25.** Subgroup analysis comparing GCF levels of resistin before and distal time after non-surgical periodontal therapy in normal-weight patients with periodontitis (NP) groups.


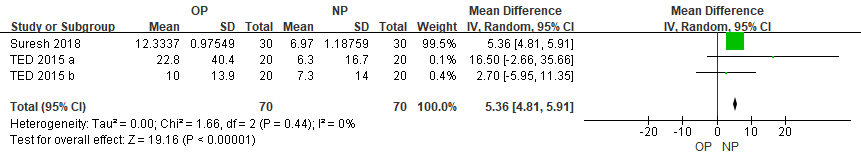


**Figure S26.** The comparing distal follow-up time GCF levels of resistin after non-surgical periodontal therapy within patients with obesity and periodontitis (OP) and normal-weight patients with periodontitis (NP) groups.


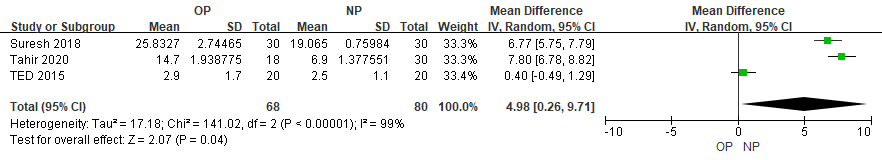


**Figure S27.** Subgroup analysis comparing baseline serum levels of resistin before non-surgical periodontal therapy within patients with obesity and periodontitis (OP) and normal-weight patients with periodontitis (NP) groups.


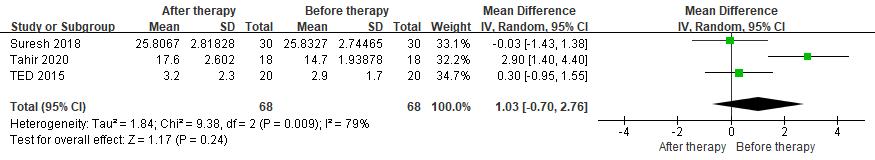


**Figure S28.** Subgroup analysis comparing serum levels of resistin before and distal time after non-surgical periodontal therapy in patients with obesity and periodontitis (OP) groups.


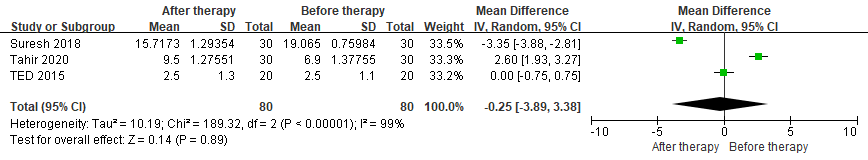


**Figure S29.** Subgroup analysis comparing serum levels of resistin before and distal time after non-surgical periodontal therapy in normal-weight patients with periodontitis (NP) groups.


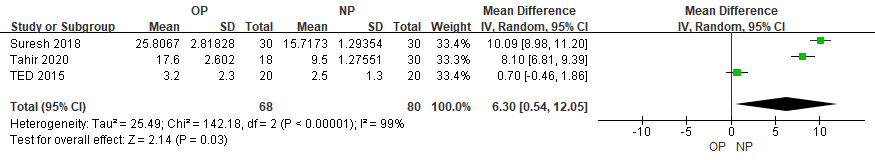


**Figure S30.** Comparison of distal follow-up time serum levels of resistin after non-surgical periodontal therapy within patients with obesity and periodontitis (OP) and normal-weight patients with periodontitis (NP) groups.


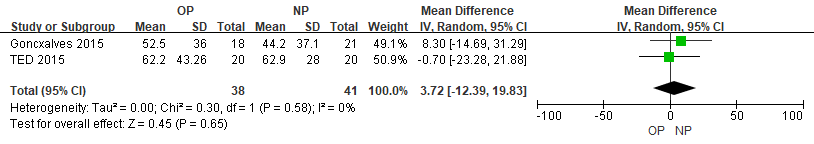


**Figure S31.** Subgroup analysis comparing baseline serum levels of adiponectin before non-surgical periodontal therapy within patients with obesity and periodontitis (OP) and normal-weight patients with periodontitis (NP) groups.


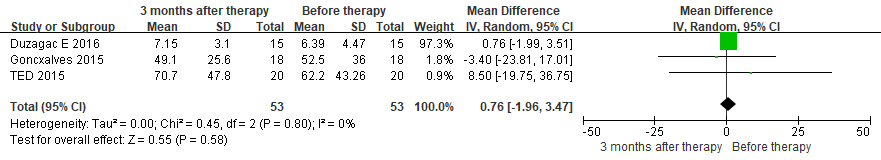


**Figure S32**. Subgroup analysis comparing serum levels of adiponectin before and three months after non-surgical periodontal therapy in patients with obesity and periodontitis (OP) groups.


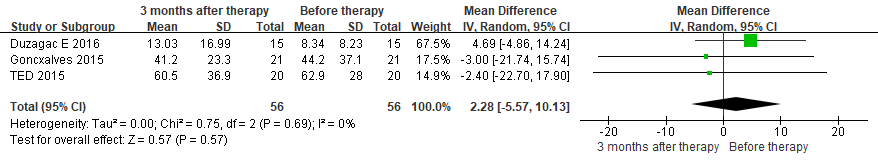


**Figure S33**. Subgroup analysis comparing serum levels of adiponectin before and three months after non-surgical periodontal therapy in normal-weight patients with periodontitis (NP) groups.


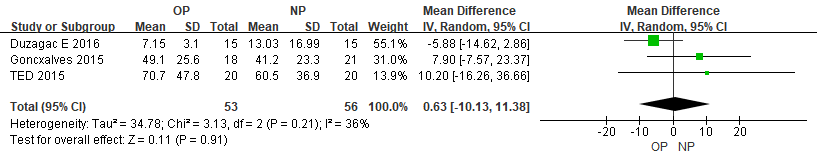


**Figure S34.** Subgroup analysis comparing 3 months follow-up serum levels of adiponectin after non-surgical periodontal therapy within patients with obesity and periodontitis (OP) and normal-weight patients with periodontitis (NP) groups.


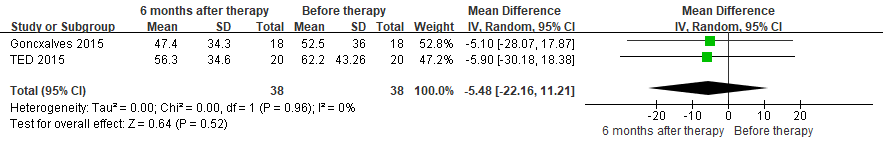


**Figure S35.** Subgroup analysis comparing serum levels of adiponectin before and six months after non-surgical periodontal therapy in patients with obesity and periodontitis (OP) groups.


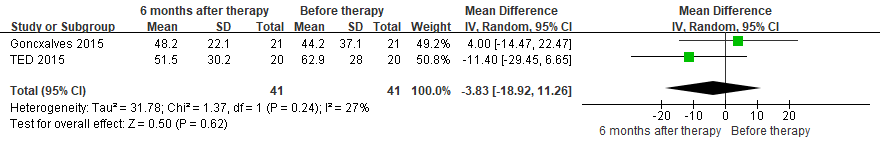


**Figure S36.** Subgroup analysis comparing serum levels of adiponectin before and six months after non-surgical periodontal therapy in normal-weight patients with periodontitis (NP) groups.


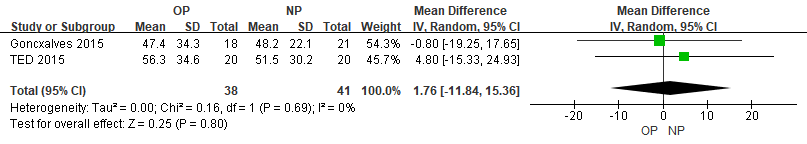


**Figure S37.** Subgroup analysis comparing 6 months follow-up serum levels of adiponectin after non-surgical periodontal therapy within patients with obesity and periodontitis (OP) and normal-weight patients with periodontitis (NP) groups.


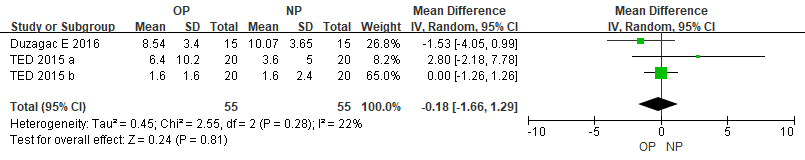


**Figure S38**. Subgroup analysis comparing baseline GCF levels of adiponectin before non-surgical periodontal therapy within patients with obesity and periodontitis (OP) and normal-weight patients with periodontitis (NP) groups.


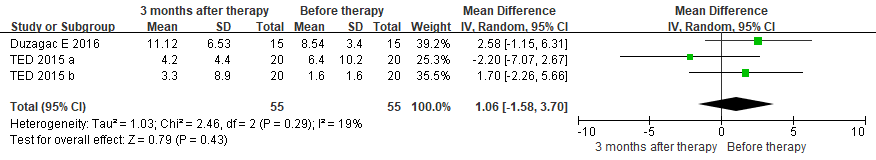


**Figure S39.** Subgroup analysis comparing GCF levels of adiponectin before and three months after non-surgical periodontal therapy in patients with obesity and periodontitis (OP) groups.


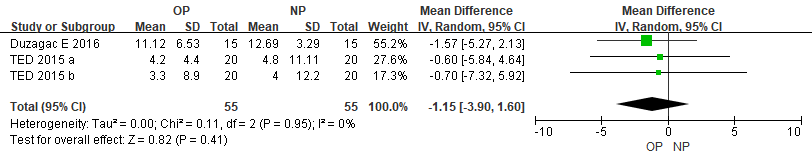


**Figure S40.** Subgroup analysis comparing 3 months follow-up GCF levels of adiponectin after non-surgical periodontal therapy within patients with obesity and periodontitis (OP) and normal-weight patients with periodontitis (NP) groups.


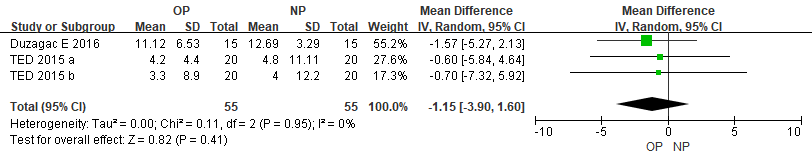


**Figure S41**. Subgroup analysis comparing baseline serum levels of leptin before non-surgical periodontal therapy within patients with obesity and periodontitis (OP) and normal-weight patients with periodontitis (NP) groups.


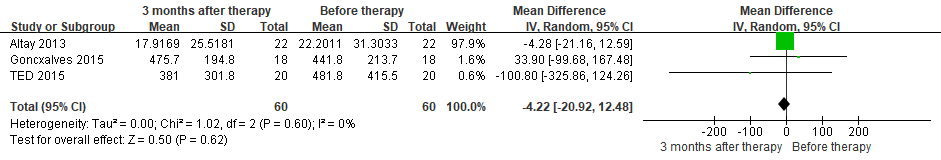


**Figure S42.** Subgroup analysis comparing serum levels of leptin before and three months after non-surgical periodontal therapy in patients with obesity and periodontitis (OP) groups.


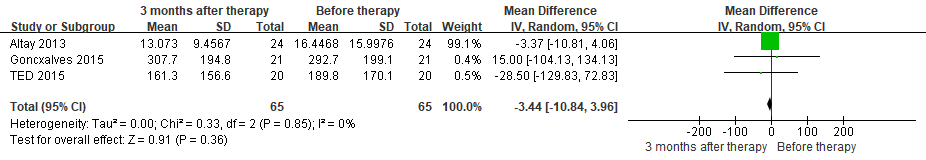


**Figure S43.** Subgroup analysis comparing serum levels of leptin before and three months after non-surgical periodontal therapy in normal-weight patients with periodontitis (NP) groups.


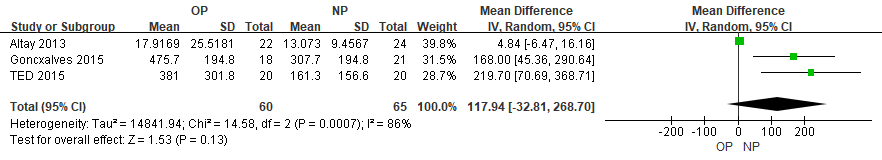


**Figure S44.** Subgroup analysis comparing 3 months follow-up serum levels of leptin after non-surgical periodontal therapy within patients with obesity and periodontitis (OP) and normal-weight patients with periodontitis (NP) groups.


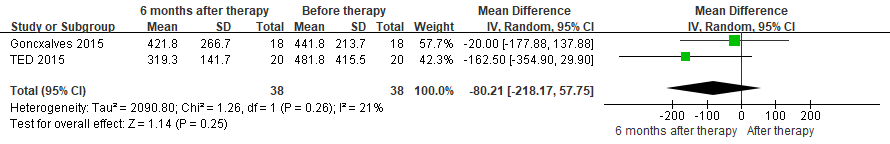


**Figure S45.** Subgroup analysis comparing serum levels of leptin before and six months after non-surgical periodontal therapy in patients with obesity and periodontitis (OP) groups.


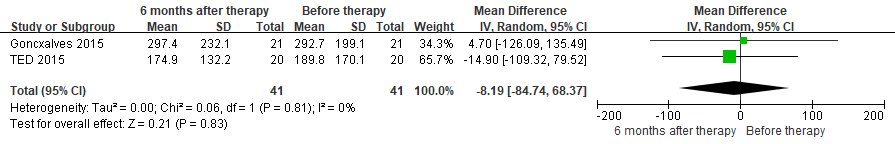


**Figure S46.** Subgroup analysis comparing serum levels of leptin before and six months after non-surgical periodontal therapy in normal-weight patients with periodontitis (NP) groups.

**References**

1. Al-Hamoudi N, Abduljabbar T, Mirza S, Al-Sowygh ZH, Vohra F, Javed F, Akram Z: **Non-surgical periodontal therapy reduces salivary adipocytokines in chronic periodontitis patients with and without obesity**. *J Investig Clin Dent* 2018, **9**(2):e12314.

2. Zuza EP, Barroso EM, Carrareto AL, Pires JR, Carlos IZ, Theodoro LH, Toledo BE: **The role of obesity as a modifying factor in patients undergoing non-surgical periodontal therapy**. *J Periodontol* 2011, **82**(5):676-682.

3. Altay U, Gürgan CA, Ağbaht K: **Changes in inflammatory and metabolic parameters after periodontal treatment in patients with and without obesity**. *J Periodontol* 2013, **84**(1):13-23.

4. Gonçalves TE, Zimmermann GS, Figueiredo LC, Souza Mde C, da Cruz DF, Bastos MF, da Silva HD, Duarte PM: **Local and serum levels of adipokines in patients with obesity after periodontal therapy: one-year follow-up**. *J Clin Periodontol* 2015, **42**(5):431-439.

5. Duzagac E, Cifcibasi E, Erdem MG, Karabey V, Kasali K, Badur S, Cintan S: **Is obesity associated with healing after non-surgical periodontal therapy? A local vs. systemic evaluation**. *J Periodontal Res* 2016, **51**(5):604-612.

6. Martinez-Herrera M, Silvestre FJ, Silvestre-Rangil J, López-Domènech S, Bañuls C, Rocha M: **Levels of serum retinol-binding protein 4 before and after non-surgical periodontal treatment in lean and obese subjects: An interventional study**. *J Clin Periodontol* 2018, **45**(3):336-344.

7. Vohra F, Akram Z, Bukhari IA, Sheikh SA, Javed F: **Short-term effects of adjunctive antimicrobial photodynamic therapy in obese patients with chronic periodontitis: A randomized controlled clinical trial**. *Photodiagnosis Photodyn Ther* 2018, **21**:10-15.

8. Çetiner D, Uraz A, Öztoprak S, Akça G: **The role of visfatin levels in gingival crevicular fluid as a potential biomarker in the relationship between obesity and periodontal disease**. *J Appl Oral Sci* 2019, **27**:e20180365.

9. Martínez-Herrera M, Abad-Jiménez Z, Silvestre FJ, López-Domènech S, Márquez-Arrico CF, Silvestre-Rangil J, Víctor VM, Rocha M: **Effect of Non-Surgical Periodontal Treatment on Oxidative Stress Markers in Leukocytes and Their Interaction with the Endothelium in Obese Subjects with Periodontitis: A Pilot Study**. *J Clin Med* 2020, **9**(7):2117.

10. Zuza EP, Barroso EM, Fabricio M, Carrareto AL, Toledo BE, J RP: **Lipid profile and high-sensitivity C-reactive protein levels in obese and non-obese subjects undergoing non-surgical periodontal therapy**. *J Oral Sci* 2016, **58**(3):423-430.

11. Suvan J, Masi S, Harrington Z, Santini E, Raggi F, D'Aiuto F, Solini A: **Effect of Treatment of Periodontitis on Incretin Axis in Obese and Nonobese Individuals: A Cohort Study**. *J Clin Endocrinol Metab* 2021, **106**(1):e74-e82.

12. Al-Zahrani MS, Alghamdi HS: **Effect of periodontal treatment on serum C-reactive protein level in obese and normal-weight women affected with chronic periodontitis**. *Saudi Med J* 2012, **33**(3):309-314.

13. Akram Z, Baharuddin NA, Vaithilingam RD, Rahim ZH, Chinna K, Krishna VG, Saub R, Safii SH: **Effect of nonsurgical periodontal treatment on clinical periodontal variables and salivary resistin levels in obese Asians**. *J Oral Sci* 2017, **59**(1):93-102.

14. Md Tahir K, Ab Malek AH, Vaithilingam RD, Saub R, Safii SH, Rahman MT, Abdul Razak F, Alabsi AM, Baharuddin NA: **Impact of non-surgical periodontal therapy on serum Resistin and periodontal pathogen in periodontitis patients with obesity**. *BMC Oral Health* 2020, **20**(1):52.

15. Suresh S, Mahendra J, Singh G, Pradeep Kumar AR, Thilagar S, Rao N: **Effect of nonsurgical periodontal therapy on plasma-reactive oxygen metabolite and gingival crevicular fluid resistin and serum resistin levels in obese and normal weight individuals with chronic periodontitis**. *J Indian Soc Periodontol* 2018, **22**(4):310-316.

16. Abdellatif HM, Ali D, Divakar DD, BinShabaib MS, SS AL: **Periodontal status and whole salivary adipokines after scaling and root planing with and without adjunct antimicrobial photodynamic therapy in obese patients with periodontitis**. *Photodiagnosis Photodyn Ther* 2022, **40**:103112.

17. Gonçalves TE, Feres M, Zimmermann GS, Faveri M, Figueiredo LC, Braga PG, Duarte PM: **Effects of scaling and root planing on clinical response and serum levels of adipocytokines in patients with obesity and chronic periodontitis**. *J Periodontol* 2015, **86**(1):53-61.
